# Carry‐over effects and fitness trade‐offs in marine life histories: The costs of complexity for adaptation

**DOI:** 10.1111/eva.13477

**Published:** 2022-09-20

**Authors:** Dustin J. Marshall, Tim Connallon

**Affiliations:** ^1^ School of Biological Sciences, and Centre for Geometric Biology Monash University Melbourne Victoria Australia

**Keywords:** Fisher's geometric model, life‐history trade‐off, pleiotropy

## Abstract

Most marine organisms have complex life histories, where the individual stages of a life cycle are often morphologically and ecologically distinct. Nevertheless, life‐history stages share a single genome and are linked phenotypically (by “carry‐over effects”). These commonalities across the life history couple the evolutionary dynamics of different stages and provide an arena for evolutionary constraints. The degree to which genetic and phenotypic links among stages hamper adaptation in any one stage remains unclear and yet adaptation is essential if marine organisms will adapt to future climates. Here, we use an extension of Fisher's geometric model to explore how both carry‐over effects and genetic links among life‐history stages affect the emergence of pleiotropic trade‐offs between fitness components of different stages. We subsequently explore the evolutionary trajectories of adaptation of each stage to its optimum using a simple model of stage‐specific viability selection with nonoverlapping generations. We show that fitness trade‐offs between stages are likely to be common and that such trade‐offs naturally emerge through either divergent selection or mutation. We also find that evolutionary conflicts among stages should escalate during adaptation, but carry‐over effects can ameliorate this conflict. Carry‐over effects also tip the evolutionary balance in favor of better survival in earlier life‐history stages at the expense of poorer survival in later stages. This effect arises in our discrete‐generation framework and is, therefore, unrelated to age‐related declines in the efficacy of selection that arise in models with overlapping generations. Our results imply a vast scope for conflicting selection between life‐history stages, with pervasive evolutionary constraints emerging from initially modest selection differences between stages. Organisms with complex life histories should also be more constrained in their capacity to adapt to global change than those with simple life histories.

## 1 INTRODUCTION

Most marine organisms have complex life histories with two or more distinct life‐history stages that can differ in habitat use, trophic mode, and mobility (Marshall et al., 2012; Strathmann, 1993). Despite years of discussion, the evolutionary forces maintaining complex life histories, and the evolutionary consequences of ecologically distinct stages, remain poorly resolved (Aguirre et al., 2014; Istock, 1967; Marshall & Morgan, 2011; Moran, 1994; Slade & Wassersug, 1975; Werner, 1988). Major life‐history transitions, such as metamorphosis, might facilitate divergence between phenotypes that are expressed at different stages during development and thereby allow individuals to express the optimal phenotype at each life‐history stage (Istock, 1967; Moran, 1994; Werner, 1988). Nevertheless, others have argued that the genetic and phenotypic links among life histories constrain evolution (Aguirre et al., 2014; Marshall & Morgan, 2011; Schluter et al., 1991; Strathmann, 1977; Wilbur, 1980). Viewed through this lens, each stage represents an awkward compromise, with trait values evolving to balance opposing patterns of selection among different life‐history stages (Schluter et al., 1991). Reconciling the degree to which different life‐history stages are constrained in their evolution is of fundamental interest but takes on new significance given the potential for such constraints to limit adaptation to environmental change. Given most marine organisms have complex histories, if life complexity constrains evolution, then marine taxa may be particularly vulnerable to global change (Marshall et al., 2016).

Quantitative genetics theory has illuminated the limits to adaptation in genetically correlated traits, including traits expressed during different life‐history stages (Lande, 1979, 1982; Marshall & Morgan, 2011). Estimates of additive genetic (co)variances and selection in multivariate trait space can predict short‐term evolutionary dynamics (Arnold, 1992; Lande, 1979), with genetic constraints to adaptation revealed through mismatches between the orientation of directional selection and the orientation of the evolutionary response (Blows & Walsh, 2009; Lande, 1979; Marshall et al., 2016). Genetic constraints that arise in simple life‐history contexts (e.g., pleiotropy between traits expressed within a stage) may be amplified by life‐history complexity. For example, if patterns of selection differ drastically between stages, even modest genetic correlations among stages can severely bias the evolutionary trajectory of each stage away from the direction favored by natural selection, and thereby reduce the rate of population adaptation (Marshall et al., 2016; Marshall & Morgan, 2011). While life‐history constraints have been assessed in several systems, the current literature is ambivalent regarding the magnitude of genetic covariances between traits expressed at different stages (Aguirre et al., 2014), as well as the consistency of natural selection across stages, with some studies showing consistent directional selection between stages and others showing reversals (Dias & Marshall, 2010; Dibattista et al., 2007; Hendry et al., 2001). But the insights that quantitative genetics theory can generate about the long‐run evolutionary trajectories of life histories are limited (see Cotto & Chevin, 2020 for a discussion of these issues)—instead a population genetics approach is required. Perhaps equally importantly, the role of carry‐over effects in altering the evolutionary trajectories of life histories has not been explored formally.

Carry‐over effects (alternatively known as latent effects or phenotypic links) occur when the environment or phenotype in one life‐history stage affects the phenotype or performance of a subsequent life‐history stage (Pechenik, 2006). For example, larval food environments can strongly influence postmetamorphic performance—high food environments generally increase juvenile survival and growth relative to low food environments (Allen & Marshall, 2010; Phillips, 2002). These effects are ubiquitous in marine taxa and play an important ecological role in driving population dynamics (Pechenik, 2006), but their role in evolution and adaptation remains surprisingly unexplored (Marshall & Morgan, 2011). By analogy, genotypes that are relatively efficient at sequestering resources early on in life may increase both their rates of survival during development and fitness components expressed later on. Here, we address these knowledge gaps by applying Fisher's geometric model to complex marine life cycles.

During the last two decades, Fisher's geometric model has emerged as a powerful tool for gauging genetic constraints to adaptation (Connallon & Hall, 2018; Connallon & Hodgins, 2021; Martin & Lenormand, 2006, 2008; Orr, 1998, 2000, 2006; Tenaillon, 2014). Several models have extended Fisher's original framework to study genetic trade‐offs in fitness between different contexts of selection, including early versus late life (Moorad & Hall, 2009), between the sexes (Connallon & Clark, 2014a, 2014b), and between environments (Connallon & Clark, 2015; Martin & Lenormand, 2006, 2015). These studies show that mutations exhibiting fitness trade‐offs may indeed be prevalent and that trade‐offs should escalate during the process of adaptation, leading to a build‐up of genetic constraints during evolution. These extensions of Fisher's model nevertheless ignore two issues that are particularly pertinent in life‐history contexts. First, their primary focus is on binary trade‐offs so the manifestation of fitness trade‐offs across life cycles with more than two major stages remains poorly characterized. Second, current theory ignores the potential for carry‐over effects between stages. While carry‐over effects potentially align the effects of mutations on performance in different stages (thereby masking trade‐offs; for example, Houle, 1991; Metcalf, 2016; van Noordwijk & de Jong, 1986), they have yet to be integrated into Fisher's geometric model.

We use Fisher's geometric model to characterize the spectrum of mutations with stage‐specific beneficial effects and the scope of genetic trade‐offs between fitness components of different life‐history stages, both with and without developmental carry‐over effects. We then consider how the pervasiveness of trade‐offs evolves during the course of adaptation to the stage‐specific optima, focusing on the evolutionary dynamics of stage‐specific viability selection and survival in populations with nonoverlapping generations. We subsequently discuss potential effects of overlapping generations on the dynamics of stage‐specific adaptation and outline areas for future theoretical attention within the framework of Fisher's geometric model.

## 2 THE MODEL

Our extension of Fisher's geometric model focuses on two key features of life‐history complexity: pleiotropy between stages, and stage differences in phenotypic selection. We ask how these features influence the emergence of genetic trade‐offs for performance among stages and constrain the trajectories of phenotypic evolution in adapting populations.

The model assumes a life cycle with *t* distinct stages (though most of our results focus on the specific cases of two or three stages, i.e.: *t* = 2 and *t* = 3), where we consider how often mutations that are beneficial with a single stage have pleiotropic costs in other stages. Using the evolutionary framework of “adaptive walks” from genetic theories of adaptation (Orr, 1998, 2005a), which applies to populations with discrete (nonoverlapping) generations, we subsequently consider how stage‐specific patterns of viability selection and phenotypic effects of random mutations affect the evolutionary trajectories of adaptation of each stage toward its optimum. Our focus on discrete‐generation dynamics reflects our primary biological interest in exploring how differences among stages in the direction of selection and the expression of genetic variation influence the evolution of trade‐offs during adaptation to an optimum, rather than maladaptation that arises from overlapping generations per se (i.e., the evolution of senescence due to age‐related declines in the efficacy of selection following reproductive maturity; Charlesworth, 1994). Evolutionary dynamics are obviously more complex in populations with overlapping generations—a point that we return to in the Discussion.

### 2.1 Fisher's geometric model with multiple stages

**FIGURE 1:**
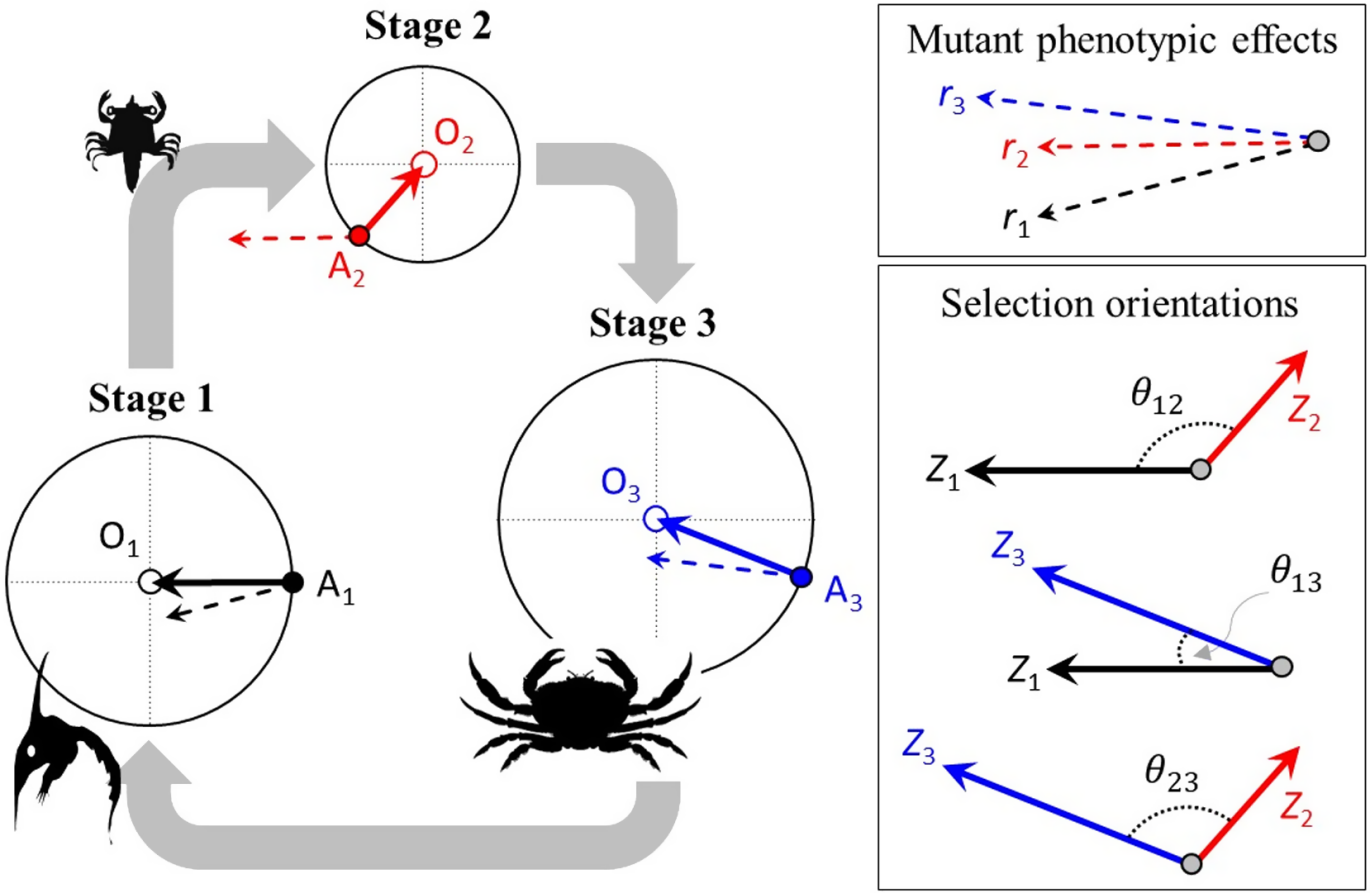
Fisher's geometric model in species with complex life cycles: an example involving three life‐history stages and two traits that are shared across stages (the traits are represented by the *y*‐ and *x*‐axes, per circle; pictograms show two different larval stages (zoea and megalopa), and the adult stage (crab). The optimal phenotype per stage (O_1_, O_2_, O_3_) occurs at the intersection between the dotted lines, and stage‐specific survival decreases with distance from the optimum. A_1_, A_2_, and A_3_ represent wild‐type phenotypes of the population (the filled circles). Directional selection in each stage is represented by a vector (the solid arrows) that points from the current phenotype to the optimal phenotype. The stage‐specific distances to the optima are represented by *z*_1_, *z*_2_, and *z*_3_. Angles between stage‐specific vectors of directional selection are $\theta_{12}$ (i.e., between stages 1 and 2), $\theta_{13}$, and $\theta_{23}$. An example is shown of a mutation that alters trait expression in all three stages. Vectors show the effect of the mutation on the phenotype with *r*_*i*_ (for stage *i*) representing the magnitude of the change. The example mutation is beneficial for stages 1 and 3 (mutant individuals are closer to the optimum) and deleterious for stage 2.

Our model builds upon the classical (i.e., haploid and “isotropic”) version of Fisher's model. Following convention (see Fisher, 1930; Kimura, 1983; Orr, 1998, 2000, 2005a, 2005b; Sellis et al., 2011; Tenaillon, 2014), we assume that: (1) individuals express multidimensional phenotypes that are entirely specified by the genotype that they carry (genotype fully specifies the phenotype expressed at each stage; the phenotypes of a given genotype may or may not vary across stages); (2) the phenotypic effects of random mutations are unbiased in multidimensional trait space; and (3) stage‐specific survival is a function of the distance between the stage‐specific phenotype and the stage‐specific optimum. With respect to assumption (1), note that random phenotypic variation among individuals with a given genotype should not affect our results, provided such variation has the same magnitude among genotypes, and the residual variation remains isotropic throughout the life cycle (see the Supporting Information). For ease of presentation, we first outline a complex life‐history extension of Fisher's geometric model in the absence of carry‐over effects between stages. We subsequently extend the model to include carry‐over effects. Figure 1 provides a two‐trait example of mutation and selection across the three stages of our model.

#### 2.1.1 Stage‐specific survival

We assume that all life‐history stages express a common set of *n* traits along with a set of *n*_*i*_ – *n* stage‐limited traits, where *n*_*i*_ represents the *total* number of traits expressed in stage *i*. Each stage has a single optimum in *n*_*i*_‐dimensional phenotypic space. The population is initially fixed for a common genotype with the following phenotypic expression values within the *i*th life‐history stage:

$$A_{i}=\left(a_{i,1},a_{i,2},\ldots ,a_{i,n},a_{i,n+1},\ldots ,a_{i,n_{i}}\right)$$

where *a*_*i*,*k*_ refers to the expression value of trait *k* during life‐history stage *i*. The first *n* terms refer to traits that are shared (mutually expressed) among stages; traits *n* + 1 to *n*_*i*_ are only expressed in the *i*th stage. The optimum phenotype for *i*th stage is:

$$O_{i}=\left(o_{i,1},o_{i,2},\ldots ,o_{i,n},o_{i,n+1},\ldots ,o_{i,n_{i}}\right)$$

with the same ordering of the *n*_*i*_ traits.

Following convention (see Orr, 1998, 2000; Tenaillon, 2014), and ignoring carry‐over effects for the moment, stage‐specific fitness declines as a Gaussian function of the Euclidean distance between trait expression and optimum. Fitness in the *i*th stage is:

$$W_{i}\left(z_{i}\right)=e^{-\omega_{i}z_{i}^{2}}$$

where *ω*_*i*_ is a constant that determines the concavity of the fitness surface in the *i*th stage, and $z_{i}=\sqrt{\sum_{k=1}^{n_{i}}{\left(o_{i,k}-a_{i,k}\right)}^{2}}$ is the distance to the optimum within the *i*th stage.

#### 2.1.2 Mutation

Each mutation alters the multivariate phenotype that is expressed by its carriers. In the *i*th stage, mutant carriers express the phenotype:

$$M_{i}=\left(a_{i,1}+\gamma_{i,1},a_{i,2}+\gamma_{i,2},\ldots ,a_{i,n_{i}}+\gamma_{i,n_{i}}\right)$$

where the *γ*_*i*,*k*_ represents the phenotypic effect of a mutation on the *k*th trait axis, in the *i*th stage.

Following Fisher (1930), Kimura (1983), Orr (1998), and others (Connallon & Clark, 2014a, 2014b; Sellis et al., 2011; Wang et al., 2010; Waxman & Welch, 2005), we assume that mutant phenotypic orientations within a given stage are unbiased in phenotypic space. For a random mutation that has a total phenotypic effect magnitude of $r_{i}=\sqrt{\sum_{k=1}^{n_{i}}\gamma_{i,k}^{2}}$ in the *i*th stage, the phenotypic orientation is generated by sampling *n*_*i*_ times (independently) from a standard normal distribution. The phenotypic effect of the mutation on the *k*th trait axis is:

(1) $$\gamma_{i,k}=\frac{r_{i}m_{i,k}}{\sqrt{\sum_{k=1}^{n_{i}}m_{i,k}^{2}}}$$

where *m*_*i*,*k*_ is the *k*th of the *n*_*i*_ drawn from the standard normal distribution. With moderately high dimensionality within the stage (*n*_*i*_ ≥ 10 is sufficient; Connallon & Clark, 2014a; Waxman & Welch, 2005), the mutation's effect on the *k*th trait axis is well‐approximated by $\gamma_{i,k}\approx r_{i}m_{i,k}/\;\sqrt{n_{i}}$, which we use in subsequent analytical results (simulations, outlined below, use the exact expression in Equation 1). We allowed mutations to have correlated phenotypic effects on each of the shared traits, with $\rho_{\mathrm{ij}}^{\mathrm{mut}}=\mathrm{cov}\left(m_{i,k},m_{i,k}\right)$ representing the covariance (between stages *i* and *j*) of random variables drawn to represent change in the *k*th of the *n* shared traits.

The displacement of mutant individuals from the optimum of the *i*th stage is $z_{i}^{*}=\sqrt{\sum_{k=1}^{n_{i}}{\left(o_{i,k}-a_{i,k}-\gamma_{i,k}\right)}^{2}}$, and the fitness (within stage *i*) of mutant relative to wild‐type individuals is:

(2) $$w_{i}=\frac{W_{i}\left(z_{i}^{*}\right)}{W_{i}\left(z_{i}\right)}=\exp\left[-\omega_{i}\left(r_{i}^{2}-2\sum_{k=1}^{n_{i}}\gamma_{i,k}\left(o_{i,k}-a_{i,k}\right)\right)\right]$$

#### 2.1.3 Evolutionary dynamics

In characterizing long‐run patterns of evolutionary change, we follow previous versions of Fisher's geometric model, and model adaptation under the assumption that mutation rates are sufficiently weak that the population may be polymorphic for, at most, a single locus at any given time (McCandlish & Stoltzfus, 2014; Orr, 1998, 2005a), and generations are nonoverlapping (for a related model of age‐specific selection with discrete generations, see Cotto & Chevin, 2020). Evolutionary dynamics can then be described using an “origin‐fixation” framework (see McCandlish & Stoltzfus, 2014), in which the population evolves through a series of discrete jumps that each correspond to the fixation of a beneficial mutation.

Fixation probabilities are based on branching process approximations (Barrett et al., 2006; Haldane, 1927a, 1927b) that emerge from the deterministic evolutionary dynamics of single loci. For a single locus with two alleles (a wild‐type and a mutant), let *q* represent the frequency of the mutant allele at the beginning of the life cycle (e.g., before any selection has occurred). In a deterministically evolving population, the change in allele frequency after a complete cycle of *t* stages is $∆q=\mathrm{sq}\left(1-q\right){\left(1+\mathrm{qs}\right)}^{-1}$ (see Supporting Information), where $s=\prod_{i=1}^{t}w_{i}-1$ represents the net fitness effect of the mutant allele (mutations with *s* > 0 experience positive selection; those with *s* < 0 experience purifying selection). Following standard theory (Barrett et al., 2006; Haldane, 1927b; Orr, 1998), the probability that a random mutation with a net fitness effect of *s* is eventually fixed is approximately $1-e^{-2s}$ when *s* > 0 and zero otherwise. Our analytical results use the approximation $1-e^{-2s}\approx 2s$, which applies with fitness effects of beneficial mutations are small (i.e., *s* < ~0.1, as is nearly always be true when the population is reasonably well adapted to its environment).

### 2.2 Carry‐over effects between stages

To include carry‐over effects, we draw upon the theory of acquisition and allocation by Houle (1991) and model stage‐specific fitness of a genotype in stage *i* as a function of its phenotypic displacement from the optimum of stage *i*, as well as the fitness of preceding stages (i.e., prior to the *i*th stage). Survival in stage *i* is:

(3) $$W_{i}\left(z\right)=\left(\prod_{j=1}^{i-1}W_{j}^{c_{\mathrm{ij}}}\right)e^{-\omega_{i}z_{i}^{2}}=\exp\left(-\omega_{i}z_{i}^{2}-\sum_{j=1}^{i-1}c_{\mathrm{ij}}\omega_{j}z_{j}^{2}\right)$$

where *c*_*ij*_ terms define the effect of survival in stage *j* on survival in stage *i*, and ***z*** = (*z*_1_, *z*_2_, … *z*_*t*_) represents the set of stage‐specific displacements from the optima. When *c*_*ij*_ = 0, there is no carry‐over effect between stage *i* and *j*; when *c*_*ij*_ > 0, high survival within the earlier stage (stage *j*) boosts survival in the later stage (stage *i*); we ignore cases where *c*_*ij*_ < 0, where poor survival in one stage increases survival in later stages as this is biologically unlikely (Pechenik, 2006). In stage *i*, the survival of a mutant relative to a wild‐type genotype is $w_{i}=W_{i}\left(z^{*}\right)/W_{i}\left(z\right)$, where $z^{*}$ represents the set of displacements from stage‐specific optima for a mutant individual.

### 2.3 Analysis and simulations

All analytical results assume that dimensionality is sufficiently high (~*n*_*i*_ > 10) that mutational effects on individual trait axes can be approximated using $\gamma_{i,k}\approx r_{i}m_{i,k}/\;\sqrt{n_{i}}$ and that mutational effects on stage‐specific survival and overall fitness are sufficiently small that fixation probabilities for beneficial mutations are 2 *s*. Analytical results were validated by exact computer simulations, which relax both assumptions.

Adaptive walk simulations were carried out under mutation‐limited conditions (see above), in which beneficial alleles are rare and adaptive substitutions fix sequentially (see McCandlish & Stoltzfus, 2014). At each time point during an adaptive walk, we generated random mutations and fitness effects using exact versions of Equations (1) and (2). For simplicity, we present the results for two extreme scenarios of the mutant phenotypic effect correlation between stages (results for intermediate cases between these extremes are presented in the Supporting Information). In the first scenario, we assumed that mutational effects are perfectly correlated between stages, and the magnitude and phenotypic orientation of each mutation were identical among stages. The magnitude of each mutation was drawn from a gamma distribution with mean *E*(*r*) = *αλ* and variance var(*r*) = *αλ* ^2^, where *α* and *λ* are the gamma shape and scale parameters (respectively). The orientation of the mutation was assigned using the algorithm presented above with $\rho_{\mathrm{ij}}^{\mathrm{mut}}=1$ (see Equation (1) and surrounding text). In the second scenario, we assumed that mutant effects are uncorrelated between stages ($\rho_{\mathrm{ij}}^{\mathrm{mut}}=0$). In this case, a mutant's magnitude was assigned for each of the stages by carrying out three independent draws from a gamma distribution and assuming identical marginal distributions for each stage.

Evolution was simulated by sequentially introducing a single mutation into the population and determining its fate by drawing a Bernoulli random variable with probability of success defined by its fixation probability ($1-e^{-2s}$, as above). Following each fixation event, we recalculated the position of the population within phenotypic space, along with new values of *z*_*i*_ and cos(*θ*_*ij*_). Parameters of the distribution of phenotypic effects of random mutations (*α*, *λ*, $\rho_{\mathrm{ij}}^{\mathrm{mut}}$), the fitness landscape of each stage (*ω*_*i*_ and the locations of optima), and trait dimensionality (*n*_*i*_), are assumed to be constant over time.

## 3 RESULTS

In the following sections, we describe four key aspects of adaptation and trade‐offs within the context of complex life cycles. We first characterize the distribution of mutational effects on stage‐specific survival. Second, we quantify the prevalence of trade‐offs in viability between life‐history stages. Third, we describe the distribution of mutational effects on overall fitness, as well as the effect of this distribution on the rate of adaptation of the population. Fourth, we describe the dynamics of stage‐specific adaptation and the escalation of trade‐offs during adaptation to a set of stage‐specific optima. For simplicity, we initially present results excluding carry‐over effects between stages. We then revisit the results in light of carry‐over effects. Because the following results are somewhat heavy going, we conclude each section (following the example of Orr, 1998) with a brief verbal summary of each section.

### 3.1 Distribution of mutational effects on stage‐specific viability

With sufficiently high phenotypic dimensions in each life stage (i.e., *n*_*i*_ > 10 in stage *i*), and conditioned on the mutation's magnitude (i.e., *r*_*i*_ in stage *i*), the distribution of mutational effects on survival in each stage converges to a multivariate normal distribution (see the Supporting Information). The mean and variance of mutational effects within the *i*th stage are given by:

(4) $$E\left[\left.\ln\left(w_{i}\right)\right|r_{i}\right]\approx -\omega_{i}r_{i}^{2},$$

and

(5) $$\mathrm{var}\left[\left.\ln\left(w_{i}\right)\right|r_{i}\right]\approx \frac{{\left(2\omega_{i}z_{i}r_{i}\right)}^{2}}{n_{i}}.$$

These expressions correspond to those of a single stage in the classical version of Fisher's geometric model (Tenaillon, 2014; Waxman & Welch, 2005). Between‐stage correlation coefficients for the effects of mutations on survival (i.e., between stages *i* and *j*, and conditioned on the mutation magnitudes, *r*_*i*_ and *r*_*j*_) are given by:

(6) $$\rho_{\mathrm{ij}}^{w}\approx \phi_{i}\phi_{j}\rho_{\mathrm{ij}}^{\mathrm{mut}}\cos\left(\theta_{\mathrm{ij}}\right).$$

Equation (6) illustrates how between‐stage correlations for survival depend on three features of mutation and selection in each pair of stages: (1) the proportion of the total maladaptation in each stage that is attributable to the shared traits ($\phi_{i}$ and $\phi_{j}$ in stages *i* and *j*, respectively, which fall within the range $0<\phi_{i},\phi_{j}<1$); (2) the correlation of phenotypic effects between the stages for the shared traits ($\rho_{\mathrm{ij}}^{\mathrm{mut}}$, which we assume is within the range ${0<\rho }_{\mathrm{ij}}^{\mathrm{mut}}<1$); and (3) the alignment of multivariate directional selection between stages for the shared traits ($\cos\left(\theta_{\mathrm{ij}}\right)$, where $\theta_{\mathrm{ij}}$ is the angle between the vectors of selection to the optimum of each stage, with respect to shared traits; $-1<\cos\left(\theta_{\mathrm{ij}}\right)<1$). There is a perfect mutational correlation for survival between stages ($\rho_{\mathrm{ij}}^{w}=1$) if (and only if) there is no maladaptation in stage‐limited traits (i.e., all maladaptation is within the shared traits: $\phi_{i}\phi_{j}=1$), phenotypic effects of mutations on shared traits are perfectly correlated between stages ($\rho_{\mathrm{ij}}^{\mathrm{mut}}=1$), and the orientations of directional selection on shared traits are perfectly aligned between stages ($\cos\left(\theta_{\mathrm{ij}}\right)=1$, which requires that $\theta_{\mathrm{ij}}=0$). Violation of the first two conditions tends to weaken the between‐stage correlation for survival; violation of the third condition will either dampen a positive correlation between stages (when $0<\cos\left(\theta_{\mathrm{ij}}\right)<1$, which requires that $\pi /2>\theta_{\mathrm{ij}}>0$), or generate a negative correlation for survival between stages ($\rho_{\mathrm{ij}}^{W}<0$ when $\cos\left(\theta_{\mathrm{ij}}\right)<0<\phi_{i}\phi_{j}\rho_{\mathrm{ij}}^{\mathrm{mut}}$).

**Message 1:** *The genetic basis of adaptive variation is likely to differ among stages under most conditions*.

### 3.2 Trade‐offs between life‐history stages

**FIGURE 2:**
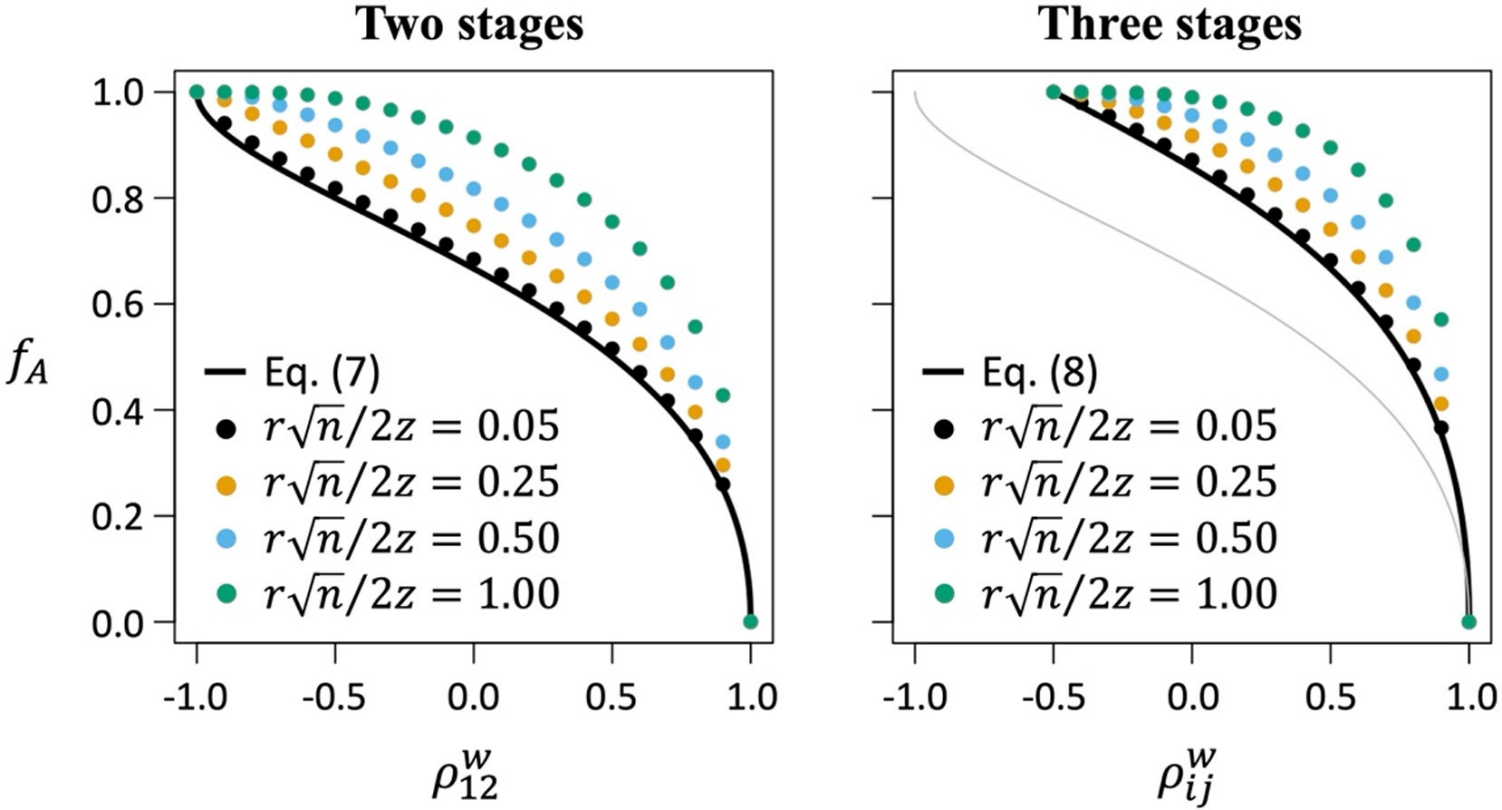
Trade‐offs between stages. Among mutations that are beneficial in at least one stage, *f*_*A*_ is the fraction that exhibits a trade‐off. The solid curves show the lower bound for *f*_*A*_ (see Equations [7 and 8]). Circles are simulated values for *f*_*A*_, each based on 10^6^ simulated mutations and different mutation sizes (mutation sizes are presented in Fisher's scale, $r\sqrt{n}/2z$, where *r* is the mutation's absolute magnitude, *n* is the number of traits, and *z* is the distance to the optimum; see Orr, 1998). Results show cases in which the mutational variance for survival (i.e., Equation 5) is equal among stages, and there are no carry‐over effects (i.e., *c*_*ij*_ = 0 in Equation (3)). In the right‐hand panel (three stages), the thin gray line shows the case where two of the three stages are perfectly correlated with each other, and the remaining stage varies in its correlation with the other two. The remaining results show the case where all stages are equally correlated with each other (i.e., $\rho_{12}^{w}=\rho_{13}^{w}=\rho_{23}^{w}$).

Imperfect correlations for survival potentially lead to genetic trade‐offs among stages, in which mutations that improve survival in some stages reduce survival in others. Among the set of mutations that increase survival in at least one life stage (hereafter, the “conditionally beneficial” mutations), what proportion exhibits trade‐offs between stages (hereafter, “*f*_*A*_”)? In the simplest case in which there are only two major stages in the life cycle (i.e., stages 1 and 2), the proportion of conditionally beneficial mutations that exhibit trade‐offs has the lower bound:

(7) $$f_{A}=\frac{2\pi -4\sin^{-1}\left(\rho_{12}^{w}\right)}{3\pi -2\sin^{-1}\left(\rho_{12}^{w}\right)}$$

(see the Supporting Information) where $\rho_{12}^{w}$ is defined in Equation (6), and sin^−1^(*x*) refers to the inverse sine function (for similar results applied to trade‐offs between sexes or between a pair of environments, see Connallon & Clark, 2014a; Martin & Lenormand, 2015). Equation (7) reveals that, unless survival is perfectly correlated between different life‐history stages ($\rho_{12}^{w}=1$), then mutations conferring increased survival in one stage will generally come at the expense of survival in another (Figure 2, left panel). Relative to the baseline defined in Equation (7), the fraction of conditionally beneficial mutations that exhibit trade‐offs further escalates with increasing mutational effect sizes (i.e., increasing sizes in Fisher's scale: $r_{i}\sqrt{n_{i}}/2z_{i}$) or when stages differ for the phenotypic effects of mutations (*r*_1_ ≠ *r*_2_), dimensionality (*n*_1_ ≠ *n*_2_), and/or their degrees of maladaptation (*z*_1_ ≠ *z*_2_).

Additional life‐history complexity further inflates the prevalence of trade‐offs among conditionally beneficial mutations. With three major stages in the life cycle, the lower bound for trade‐offs becomes:

(8) $$f_{A}=\frac{6\pi -4\left(\sin^{-1}\left(\rho_{12}^{w}\right)+\sin^{-1}\left(\rho_{13}^{w}\right)+\sin^{-1}\left(\rho_{23}^{w}\right)\right)}{7\pi -2\left(\sin^{-1}\left(\rho_{12}^{w}\right)+\sin^{-1}\left(\rho_{13}^{w}\right)+\sin^{-1}\left(\rho_{23}^{w}\right)\right)},$$

(see the Supporting Information). Trade‐offs are equally prevalent in two‐ and three‐stage life cycles when, at most, one life‐history stage is imperfectly correlated with the other(s) (e.g., $\rho_{12}^{w}=\rho_{13}^{w}<1$ and $\rho_{23}^{w}=1$), in which the system behaves as if it is composed of effectively two stages (Equation (8) reduces to Equation (7)). Trade‐offs escalate when all three stages are imperfectly correlated with one other (e.g., $-0.5<\rho_{12}^{w}=\rho_{13}^{w}=\rho_{23}^{w}<1$; see Figure 2, right panel).

**Message 2:** *Trade‐offs in survival between life‐history stages are almost inevitable when there is pleiotropy between traits expressed in different stages*. *The prevalence of these trade‐offs is expected to increase with the number of life‐history stages in the life cycle*.

### 3.3 Beneficial mutations and the rate of adaptation

Adaptation is fueled by beneficial alleles that improve the overall fitness of their carriers. In the absence of carry‐over effects, and with nonoverlapping generations, the relative fitness of a random mutation is a multiplicative function of its effects on fitness components (e.g., probabilities of survival) for the set of life‐history stages (i.e.: $1+s=\prod_{i=1}^{t}w_{i}$, where *s* is the net selection coefficient for the mutation, *t* denotes the number of stages in the life cycle, and the *w*_*i*_ are described in Equation (2)). At high dimensions (*n*_*i*_ > 10) and provided values of *s* are small (|*s*| << 1, and therefore ln[1 + *s*] ~ *s*), the distribution of fitness effects of mutations of size ***r*** = (*r*_1_, *r*_2_, …, *r*_*t*_) is approximately normal with a mean and variance (respectively) of:

(9) $$\bar{s}=-\sum_{i=1}^{t}\omega_{i}r_{i}^{2},$$

and

(10) $$\sigma^{2}=\sum_{i=1}^{t}\frac{{\left(\beta_{i}r_{i}\right)}^{2}}{n_{i}}+2\sum_{i=1}^{t}\sum_{j>1}^{t}\frac{\beta_{i}\beta_{j}r_{i}r_{j}}{\sqrt{n_{i}n_{j}}}\rho_{\mathrm{ij}}^{w},$$

where $\beta_{i}=2\omega_{i}z_{i}$ is the strength of selection to the optimum in stage *i*. Equation (10) implies that the variance of the distribution of fitness effects is proportional to the mutational correlations for survival between each pair of stages. The variance is maximized when $\rho_{\mathrm{ij}}^{w}=1$, which—as we have seen—is a restrictive condition to meet. Following Cotto and Chevin (2020), when constraints to divergence between stages are absolute and trait expression is constant across the life cycle (***A*** = ***A***_***i***_; *n* = *n*_*i*_, *r* = *r*_*i*_; $\rho_{\mathrm{ij}}^{\mathrm{mut}}=1$), then the model can be reframed in terms selection to an “effective optimum,” which represents a phenotypic compromise that minimizes the cumulative cost of maladaptation through the life cycle (see the Supporting Information). In this case, Equations (9 and 10) simplify to $\bar{s}=-\omega_{\mathrm{tot}}r^{2}$ and $\sigma^{2}\approx n^{-1}{\left(2\tilde{z}r\omega_{\mathrm{tot}}\right)}^{2}$, where $\tilde{z}$ is the population's distance from the effective optimum and $\omega_{\mathrm{tot}}=\sum_{i=1}^{t}\omega_{i}$.

Imperfect correlations between stages ($\rho_{\mathrm{ij}}^{w}<1$) decrease the rate of adaptation of the population. Assuming that adaptation is mutation‐limited and dimensionality is reasonably large (*n*_*i*_ > 10), the expected rate of adaptation (increase in relative fitness) via fixation of mutations of size ***r*** is:

(11) $$\frac{d\ln\left(\left.w\right|r\right)}{\mathrm{dt}}=\mathrm{Nu}\sigma^{2}\left[\left(1+\frac{{\bar{s}}^{2}}{\sigma^{2}}\right)\left(1+\mathrm{erf}\left(\frac{\bar{s}}{\sigma \sqrt{2}}\right)\right)+\frac{\bar{s}}{\sigma }\frac{\sqrt{2}}{\sqrt{\pi }}e^{-{\bar{s}}^{2}/2\sigma^{2}}\right]$$

(see the Supporting Information), where $\bar{s}$ and $\sigma^{2}$ are given by Equations (9 and 10), *Nu* is the population‐scaled mutation rate, and erf$\left(\frac{\bar{s}}{\sigma \sqrt{2}}\right)$ is the error function. Equation (11), which follows the approach pioneered by Orr, 2000; (also see Tenaillon, 2014; Connallon & Hall, 2018), is a monotonically increasing function of genetic correlations of survival among life‐history stages (i.e., $\frac{d}{d\rho_{\mathrm{ij}}^{w}}\left(\frac{d\ln\left(\left.w\right|r\right)}{\mathrm{dt}}\right)>0$). In other words, decreasing values of $\rho_{\mathrm{ij}}^{w}$ reduce the rate at which the population can adapt to its environment. In the extreme case, in which the variance for survival is equal among stages (Equation (5) is the same for each stage) and correlations in survival reach the common lower bound, $\rho_{\mathrm{ij}}^{w}=-{\left(t-1\right)}^{-1}$, beneficial effects of mutations are offset by costs in other stages, which minimizes the mutational variance for fitness ($\sigma^{2}=0$), renders mutations effectively neutral, and causes adaptation to cease.

**Message 3:** *Species with complex life histories will adapt much more slowly than those with simple life histories*. *Mutations can become effectively neutral with respect to fitness despite affecting survival of each stage of the life cycle*.

### 3.4 Stage‐specific adaptation and the escalation of constraints during evolution

How do imperfectly correlated effects of mutations on survival across stages affect the long‐run evolutionary trajectories of each stage toward its optimum? How does the process of adaptation influence the emergence of evolutionary genetic constraints over time? We answer these questions using simulated adaptive walks of populations with three major life‐history stages to a set of stage‐specific phenotypic optima. We focused on two extremes of our model. At the one extreme, phenotypic effects of mutations are perfectly correlated between stages: all traits are shared, and mutational magnitudes and orientations are perfectly correlated among stages (*r*_*i*_ = *r*_*j*_, $\rho_{\mathrm{ij}}^{\mathrm{mut}}=1$). At the other extreme, mutational effects are uncorrelated between stages (cov(*r*_*i*_, *r*_*j*_) = 0, $\rho_{\mathrm{ij}}^{\mathrm{mut}}=0$), though mutations have pleiotropic effects across stages. For simplicity, we assumed that stages exhibit similar marginal distributions of mutational effects on trait expression (*r*_*i*_ and *r*_*j*_ have the same marginal distribution in stages *i* and *j*, respectively), and stages have similar strengths of stabilizing selection near their optima (i.e., *ω*_*i*_ = *ω*_*j*_).

**FIGURE 3:**
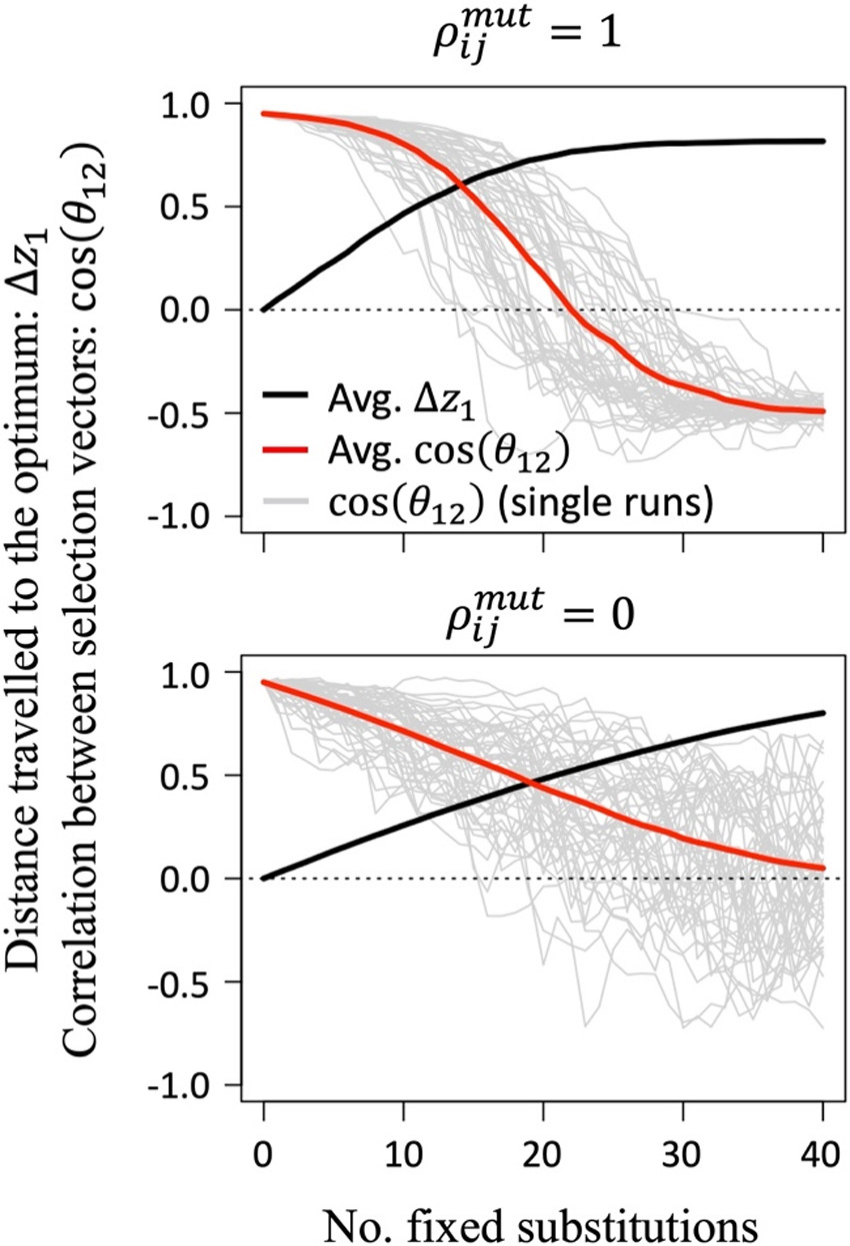
Stage‐specific adaptation and orientations of directional selection during adaptive walks toward stage‐specific optima. Results show cases where the optima for a life cycle of three stages are equally divergent from one another (they form an equilateral triangle in multidimensional space) and there are no carry‐over effects (*c*_12_ = *c*_13_ = *c*_23_ = 0). Given symmetry in these results, we present the evolutionary movement of the first stage to its optimum ($∆z_{1}$, where $∆z_{1}=0$ corresponds to no adaptation, and $∆z_{1}=1$ corresponds to a completed adaptive walk), and the orientation of selection in stage 1 relative to stage 2 ($-1<\cos\left(\theta_{12}\right)<1$, where $\cos\left(\theta_{12}\right)$ captures the correlation of directional selection between stages 1 and 2). Means are based on 100 adaptive walks (top panel) or 5000 walks (bottom panel), with gray lines showing the values of $\cos\left(\theta_{12}\right)$ for the first 50 adaptive walks. Initial conditions were *z*_1_ = *z*_2_ = *z*_3_ = 1 and cos(*θ*_12_) = cos(*θ*_13_) = cos(*θ*_23_) = 0.95, with selection parameters *ω*_1_ = *ω*_2_ = *ω*_3_ = ½, and equal marginal distributions of mutant phenotypic effects on each stage. Mutational magnitudes (*r*_1_, *r*_2_, *r*_3_) follow an exponential distribution (gamma with parameters *α* = 1 and *λ* = 0.1). Results for intermediate phenotypic effect correlations between stages are presented in Figure S2.

When mutant phenotypic effects are strongly correlated across stages, any initial differences among stages in the orientation of directional selection become amplified during the process of adaptation. In a three‐stage model with the optima equidistant from each other (Figure 3, top panel), adaptive substitutions tend to increase the angles between stage‐specific vectors of directional selection (i.e., *θ*_*ij*_ increases and cos(*θ*_*ij*_) decreases), which decreases mutational correlations for survival and expands the prevalence of trade‐offs between stages (i.e., given Equation (8)). When all traits are shared (*n*_1_ = *n*_2_ = *n*_3_ = *n*), mutational correlations for survival ($\rho_{\mathrm{ij}}^{w}$) ultimately approach a limit at which $\rho_{12}^{w}=\rho_{13}^{w}=\rho_{23}^{w}=-0.5$ and no further adaptation is possible (Figure 3, top panel). When the optimum of one stage is divergent from the others and stabilizing selection is equally strong for each stage, adaptation becomes enhanced in stages with similar optima at the expense of the stage with the most divergent optimum (see Figures S1 and S2). In the extreme case where two stages share an optimum and the remaining optimum is divergent, the system tends to evolve to a state of constraint in which the sum of the displacements of the two similar stages are equal the displacement of the most divergent stage.

When mutant phenotypic effects are uncorrelated across stages, trade‐offs are common regardless of the degree to which directional selection aligns between stages (i.e., from Equation (8), $f_{A}\geq 6/7\approx 0.86$ when $\rho_{\mathrm{ij}}^{\mathrm{mut}}=0$ and there are three stages). Our simulations further show that any alignment among stages in the initial direction of selection will tend to decay over time; on average, the angles between vectors of stage‐specific selection evolve to become orthogonal (*θ*_*ij*_ = *π*/2 and cos(*θ*_*ij*_) = 0), though there is considerable dispersion across different adaptive walk simulation runs in the relative orientations of stage‐specific selection (cf. the mean value for cos(*θ*_*ij*_), presented in red, versus cos(*θ*_*ij*_) of individual simulation runs, presented in gray; Figure 3, bottom panel). All stages will eventually approach their optima, though rates of adaptation can be slow due to the prevalence of pleiotropic trade‐offs among stages.

**Message 4:** *Maladaptation persists in each stage and is particularly pronounced in the stage with the most divergent optimum*. *Selection will eventually resolve this maladaptation over time*.

### 3.5 Evolutionary consequences of carry‐over effects between stages

Up to this point, we have ignored developmental carry‐over effects between stages, thereby excluding the likely possibility that high survival in early stages (e.g., high rates of resource acquisition; Houle, 1991; Marshall & Morgan, 2011; Pechenik, 2006; van Noordwijk & de Jong, 1986) will benefit survival at later ones. In the presence of carry‐over effects (following Equation (3)), stage‐specific survival in each of the three stages is described by the following functions: $W_{1}=e^{-\omega_{1}z_{1}^{2}}$, $W_{2}=W_{1}^{c_{12}}e^{-\omega_{2}z_{2}^{2}}$, and $W_{3}=W_{1}^{c_{13}}W_{2}^{c_{23}}e^{-\omega_{3}z_{3}^{2}}$, with the superscripts characterizing the carry‐over effects of survival from earlier to later stages (*c*_*ij*_ = 0 when there are no carry‐over effects; *c*_*ij*_ >0 otherwise).

Carry‐over effects between stages promote positive between‐stage covariances for survival and thereby limit the expression of trade‐offs in survival between stages. The effect is easily seen for the case of two stages, where the covariance of mutational effects on survival in stages 1 and 2 (conditional on the mutational magnitudes, *r*_1_ and *r*_2_) is:

(12) $$\mathrm{cov}\left[\left.\ln\left(w_{1}\right),\ln\left(w_{2}\right)\right|r_{1},r_{2}\right]\approx \frac{4\omega_{1}\omega_{2}r_{1}r_{2}z_{1}z_{2}}{\sqrt{n_{1}n_{2}}}\phi_{1}\phi_{2}\rho_{12}^{\mathrm{mut}}\cos\left(\theta_{12}\right)+c_{12}{\left(\frac{2\omega_{1}r_{1}z_{1}}{\sqrt{n_{1}}}\right)}^{2},$$

where $\ln\left(w_{1}\right)$ and $\ln\left(w_{2}\right)$ represent the survival of mutant relative to wild‐type individuals in stages 1 and 2, respectively. The second term in the right‐hand side of the equation captures the contribution of carry‐over effects to the covariance. This term is positive whenever carry‐over effects are present (*c*_*ij*_ > 0), and will either enhance an already positive covariance for survival between stages (i.e., when $\phi_{1}\phi_{2}\rho_{12}^{\mathrm{mut}}\cos\left(\theta_{12}\right)>0$) or weaken a negative covariance between stages (i.e., when $\phi_{1}\phi_{2}\rho_{12}^{\mathrm{mut}}\cos\left(\theta_{12}\right)<0$). When phenotypic effects of mutations are uncorrelated between stages ($\rho_{12}^{\mathrm{mut}}=0$), carry‐over effects will, nevertheless, generate a positively correlation for survival in each stage.

**FIGURE 4:**
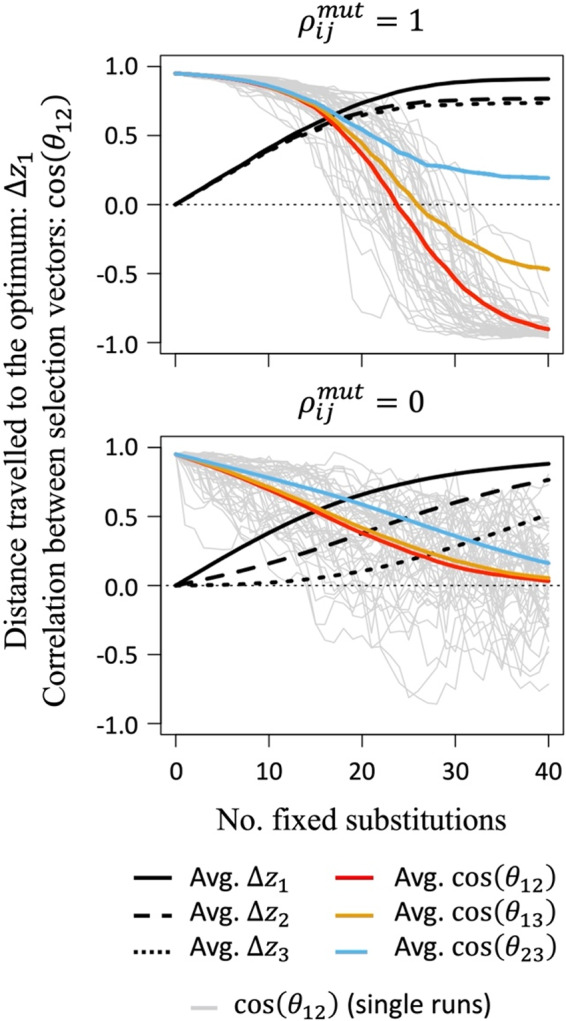
Phenotypic divergence and stage‐specific orientations of selection during adaptive walks with strong carry‐over effects between stages (*c*_12_ = *c*_13_ = *c*_23_ = 2). The simulations are otherwise identical to those in Figure 3. Results for intermediate phenotypic effect correlations between stages are presented in Figure S2.

Adaptive walk simulations reveal an additional consequence of carry‐over effects for stage‐specific patterns of adaptation. Because survival in earlier stages has cascading effects on survival throughout the remainder of the life cycle, carry‐over effects tip the balance of adaptation in the favor of earlier stages, and at the expense of later ones (Figure 4), with early stages more closely approaching their optima than later stages. Carry‐over effects cause maladaptation to increase with each successive stage of the life cycle, with the relative orientations of selection becoming most similar between the later, more poorly adapted stages (Figure 4).

**Message 5**: *Carry‐over effects tend to reduce survival trade‐offs between stages and give an advantage to earlier life‐history traits in the evolutionary tug‐of‐war over survival in each life‐history stage*.

## 4 DISCUSSION

Our results suggest that genetic trade‐offs in survival among life‐history stages should be ubiquitous in organisms with complex life histories and that rates of adaptation may, therefore, be dampened relative to organisms with simple life histories. Evolutionary theory has long emphasized the constraints to adaptation that naturally emerge from pleiotropy between the traits expressed by each organism (Fisher, 1930; Orr, 1998, 2000; but see Wang et al., 2010). Life‐history complexity, and pleiotropy between the distinct stages of a life cycle, further escalates these constraints. Simply put, all else being equal a species with numerous life‐history stages (e.g., a multi‐host helminth parasite or a jellyfish) may be more constrained in their evolution than a species with a simpler life history (e.g., a shark with direct development). Carry‐over effects can partially alleviate these constraints by reducing the scope of fitness trade‐offs between stages, but genetic trade‐offs are still likely to be prevalent during the process of adaptation, particularly when the phenotypic effects of mutations are strongly correlated among stages. Surprisingly, carry‐over effects shift the balance in favor of early life‐history stages such that higher survival early in the life‐history will arise at the expense of survival in later life‐history stages.

### 4.1 Conflicting selection between stages as a consequence of adaptive evolution

Trade‐offs are virtually inevitable when mutations have pleiotropic phenotypic effects across stages. Indeed, only under restrictive circumstances—perfect correlations of mutant phenotypic effects between stages, and perfect alignment of stage‐specific directional selection—will complex life histories be free from trade‐offs. Previous discussions of evolutionary constraints in complex life histories have failed to anticipate the acute sensitivity of performance trade‐offs to such minor misalignments in selection (Moran, 1994; but see Marshall & Morgan, 2011). Interestingly, larval biologists have long observed evidence of maladaptation in early life‐history stages, including the observation that developmental failure is common (not all eggs develop into larvae and not all larvae metamorphose into juveniles [Strathmann, 1987]). While some of this developmental failure is likely a product of imperfect culture conditions, our model implies that some failure could be genetically hardwired and an unavoidable by‐product of adaptation in other life‐history stages.

Our findings predict a surprising association between adaptation of the population and misalignment of directional selection between stages. As we show above, the process of adaptation leads to divergence in the orientations of directional selection in each life‐history stage. As a result, conflict escalates over time, even in cases when directional selection is initially strongly aligned among the stages. Divergent selection among stages may arise from (perhaps slight) initial differences in the orientations of displacement of each stage from its optimum, or because mutations contributing to adaptation differentially affect the phenotypes expressed at each stage, which can generate divergent displacements from a shared optimum (also see Connallon & Clark, 2014a; Martin & Lenormand, 2015). Paradoxically, if we observe high alignment of selection among stages in nature, this may imply that the population is poorly adapted to its current environment—the high alignment of selection among stages could reflect the population's distance from evolutionary equilibrium. In contrast, misaligned selection among stages may indicate that the population is not far from evolutionary equilibrium and is relatively well adapted, despite suboptimal expression of the phenotypes expressed at each stage.

The degree to which directional selection across stages aligns may provide clues to whether the population has recently experienced environmental change. For example, populations in which directional selection strongly aligns among stages are likely to reflect recent or persistent environmental change to which the population has not yet adapted. These nonequilibrium populations may yet evolve trade‐offs between stages if environmental conditions stabilize in future. These theoretical predictions closely parallel those from the sexual conflict literature, in which environmental change appears to mediate genetic trade‐offs between female and male fitness (Berger et al., 2014; Connallon, 2015; Connallon & Hall, 2016; Long et al., 2012). An interesting challenge for empiricists would be to determine whether populations at the edge of species' range expansion show more alignment in selection between, say, larval and adult phases, than populations at the core of a species range (which are presumably better adapted to those conditions).

### 4.2 Life‐history complexity and the optimization of stage‐specific phenotypes

Given the exquisite array of adaptations expressed by larval and adult individuals of a species, it has always been tempting to interpret the phenotypes that are expressed during each stage as being optimized for stage‐specific survival. The phenotypic differences between stages surely reflect the differences in selection between them. However, our model illustrates that there remains considerable scope for a tug‐of‐war between pleiotropically‐linked stages that may drag stage‐specific phenotypes in nonadaptive directions within multivariate trait space. Thus, a trait in any one life‐history stage may actually be a by‐product of selection in another stage. At the very least, evolution in one stage should often alter the phenotypes that can evolve in others, particularly when pleiotropy and/or carry‐over effects maintain phenotypic links among stages.

Adaptation in complex life histories is surprisingly fraught with conflict, with slight misalignments in selection between stages rapidly yielding rampant genetic trade‐offs. When selection is imperfectly aligned across stages (perfect alignment in selection is unlikely to occur and even less likely to persist across life histories; Marshall & Keough, 2008; Pettersen et al., 2016), the evolutionary response of a single trait or stage to selection is expected to decrease with increasing numbers of life‐history stages. This reduction in the efficacy of stage‐specific selection arises as a natural by‐product of selection discordance between stages.

Carry‐over effects ameliorate the pervasiveness of genetic trade‐offs among life‐history stages, but preserve the conflicts between stages that emerge during adaptation. Interestingly, we find that carry‐over effects tend to skew evolution in favor of the early life‐history stages (e.g., larval) at the expense of later ones (e.g., adult). From this perspective, the evolution of adult traits should be subordinate to the evolution of larval traits. All else being equal, larval stages should be better adapted to their environment than are adult stages. Survival of larvae is also more sensitive to environmental stressors than survival of adults (Marshall et al., 2016), which may imply that selection toward the optimum within the larval stage may typically be stronger than selection within the adult stage (in our model, this corresponds to a larger *ω* parameter in the larval relative to the adult stage). If true, the combination of intrinsically stronger selection at the larval stage, and a carry‐over effect, would ensure that larvae remain closer to their optimum than adults are to theirs. Carry‐over effects should also add to the maladaptation of postreproductive stages that arise through senescence, and which also amplify maladaptation in adults relative to juveniles (see Cotto & Ronce, 2014; Moorad & Promislow, 2008). The ecological consequences of carry‐over effects are relatively well understood—that they play an important evolutionary role has been less appreciated until now.

Overall, our results strongly suggest that organisms with complex life histories are likely to show less capacity to adapt to environmental change than organisms with simple life histories. We find that the more life‐history stages, the more constrained evolution is likely to be. This finding has particularly worrying implications for organisms such as red alga and multi‐host helminth parasites with more than 5 distinct life‐history stages—our model implies adaptation to change will be particularly constrained in such groups. Conversely, species with simple life histories (e.g., direct developers) should, ceteris paribus, be more robust to environmental change. A better understanding of the genetic covariances among life‐history stages is essential if we are to predict how marine organisms are likely to cope with future change. There is a temptation to view any one life‐history stage as well adapted to its environment, yet our results suggest that any one stage (and particularly the adult stage) is likely to represent an awkward compromise between constraints imposed by other stages and the selective forces acting on that stage (also see Cotto et al., 2019).

Finally, it is important to recall an important simplifying assumption of our model: that generations are nonoverlapping. As such, our predictions directly apply to some marine species (e.g., short‐lived, weedy species and taxa exhibiting semelparous reproduction; for relevant discussion, see Cotto & Chevin, 2020) but many not apply as well to others (e.g., iteroparous species). Classical population genetic models of age‐structured populations (Charlesworth, 1994) suggest two ways in which overlapping generations should alter stage‐specific evolutionary dynamics relative to our predictions. First, whereas adaptation in our model favors mutations that increase the net probability that individuals survive to reproduce, models with overlapping generations should favor mutations that elevate population growth, at least to a reasonable approximation when mutations have small effects on viability and/or fertility (Charlesworth, 1994, p. 179; Haldane, 1927a; but note that this rule of thumb can break down when beneficial mutations have strong effects on fitness components; see de Vries & Caswell, 2019). Second, the efficacy of selection with overlapping generations declines with age following reproductive maturity, which should differentially hinder adaptation in late relative to early stages of the life cycle. Consequently, our prediction that carry‐over effects will amplify maladaptation in late stages is likely to be conservative in taxa with overlapping generations, which may suffer even greater levels of maladaptation owing to processes contributing senescence (Moorad & Promislow, 2008). A complete description of the population genetics of adaptation to an optimum (Orr, 1998, 2005a, 2005b), including effects of overlapping generations, requires an expanded analytical framework that explicitly combines evolution and age‐structured demographic dynamics (see Barfield et al., 2011; Cotto et al., 2019; de Vries & Caswell, 2019; Orive et al., 2017), which is a worthy topic for future theoretical work.

## CONFLICT OF INTEREST

The authors declare no conflicts of interest.

## Supporting information


Figures S1‐S2

## Data Availability

Theory paper, no data.

## References

[eva13477-bib-0001] Aguirre, J. D. , Blows, M. W. , & Marshall, D. J. (2014). The genetic covariance between life cycle stages separated by metamorphosis. Proceedings of the Royal Society B: Biological Sciences, 281(1788), 20141091. 10.1098/rspb.2014.1091 PMC4083807 24966319

[eva13477-bib-0002] Allen, R. M. , & Marshall, D. J. (2010). The larval legacy: cascading effects of recruit phenotype on post‐recruitment interactions. Oikos, 119(12), 1977–1983.

[eva13477-bib-0003] Arnold, S. J. (1992). Constraints on phenotypic evolution. The American Naturalist, 140, S85–S107. 10.1086/285398 19426028

[eva13477-bib-0004] Barfield, M. , Holt, R. D. , & Gomulkiewicz, R. (2011). Evolution in stage‐structured populations. The American Naturalist, 177(4), 397–409. 10.1086/658903 PMC5016196 21460563

[eva13477-bib-0005] Barrett, R. D. H. , M'Gonigle, L. K. , & Otto, S. P. (2006). The distribution of beneficial mutant effects under strong selection. Genetics, 174(4), 2071–2079. 17028334 10.1534/genetics.106.062406 PMC1698630

[eva13477-bib-0006] Berger, D. K. , Grieshop, M. I. , Lind, J. , Goenaga, A. A. , Maklakov, G. , & Arnqvist . (2014). Intralocus sexual conflict and environmental stress. Evolution, 68(8), 2184–2196. 24766035 10.1111/evo.12439

[eva13477-bib-0007] Blows, M. , & Walsh, B. (2009). Spherical cows grazing on flatland: constraints to selection and adaptation. In J. Van der Werf (Eds.), Adaptaton and fitness in animal populations (pp. 83–101). Springer.

[eva13477-bib-0008] Charlesworth, B. (1994). Evolution in age‐structured populations (2nd ed.). Cambridge University Press.

[eva13477-bib-0009] Connallon, T. (2015). The geography of sex‐specific selection, local adaptation, and sexual dimorphism. Evolution, 69(9), 2333–2344. 26194274 10.1111/evo.12737

[eva13477-bib-0010] Connallon, T. , & Clark, A. G. (2014a). Evolutionary inevitability of sexual antagonism. Proceedings of the Royal Society B: Biological Sciences, 281(1776), 20132123. 10.1098/rspb.2013.2123 PMC3871308 24335980

[eva13477-bib-0011] Connallon, T. , & Clark, A. G. (2014b). Balancing selection in species with separate sexes: insights from Fisher's geometric model. Genetics, 197(3), 991–1006. 24812306 10.1534/genetics.114.165605 PMC4096376

[eva13477-bib-0012] Connallon, T. , & Clark, A. G. (2015). The distribution of fitness effects in an uncertain world. Evolution, 69(6), 1610–1618. 25913128 10.1111/evo.12673 PMC4716676

[eva13477-bib-0013] Connallon, T. , & Hall, M. D. (2016). Genetic correlations and sex‐specific adaptation in changing environments. Evolution, 70(10), 2186–2198. 27477129 10.1111/evo.13025

[eva13477-bib-0014] Connallon, T. , & Hall, M. D. (2018). Genetic constraints on adaptation: a theoretical primer for the genomics era. Annals of the New York Academy of Sciences, 1422(1), 65–87. 29363779 10.1111/nyas.13536

[eva13477-bib-0015] Connallon, T. , & Hodgins, K. A. (2021). Allen Orr and the genetics of adaptation. Evolution, in press, 75, 2624–2640. 34606622 10.1111/evo.14372

[eva13477-bib-0016] Cotto, O. , & Chevin, L. M. (2020). Fluctuations in lifetime selection in an autocorrelated environment. Theoretical Population Biology, 134, 119–128. 32275919 10.1016/j.tpb.2020.03.002 PMC7115991

[eva13477-bib-0017] Cotto, O. , & Ronce, O. (2014). Maladaptation as a source of senescence in habitats variable in space and time. Evolution, 68(9), 2481–2493. 24909756 10.1111/evo.12462

[eva13477-bib-0018] Cotto, O. , Sandell, L. , Chevin, L. M. , & Ronce, O. (2019). Maladaptive shifts in life history in a changing environment. The American Naturalist, 194(4), 558–573. 10.1086/702716 31490719

[eva13477-bib-0019] de Vries, C. , & Caswell, H. (2019). Stage‐structured evolutionary demography: linking life histories, population genetics, and ecological dynamics. The American Naturalist, 193(4), 545–559. 10.1086/701857 30912967

[eva13477-bib-0020] Dias, G. M. , & Marshall, D. J. (2010). Does the relationship between offspring size and performance change across the life‐history? Oikos, 119(1), 154–162.

[eva13477-bib-0021] Dibattista, J. D. , Feldheim, K. A. , Gruber, S. H. , & Hendry, A. P. (2007). When bigger is not better: selection against large size, high condition and fast growth in juvenile lemon sharks. Journal of Evolutionary Biology, 20(1), 201–212. 17210013 10.1111/j.1420-9101.2006.01210.x

[eva13477-bib-0022] Fisher, R. A. (1930). The genetical theory of natural selection. Oxford University Press.

[eva13477-bib-0023] Haldane, J. B. S. (1927a). A mathematical theory of natural and artificial selection, Part IV. Proceedings of the Cambridge Philosophical Society, 23(5), 607–615.

[eva13477-bib-0024] Haldane, J. B. S. (1927b). A mathematical theory of natural and artificial selection, Part V: selection and mutation. Proceedings of the Cambridge Philosophical Society, 23(7), 838–844.

[eva13477-bib-0025] Hendry, A. P. , Day, T. , & Cooper, A. B. (2001). Optimal size and number of propagules: Allowance for discrete stages and effects of maternal size on reproductive output and offspring fitness. The American Naturalist, 157(4), 387–407. 10.1086/319316 18707249

[eva13477-bib-0026] Houle, D. (1991). Genetic covariance of fitness correlates: what genetic correlations are made of and why it matters. Evolution, 45(3), 630–648. 28568816 10.1111/j.1558-5646.1991.tb04334.x

[eva13477-bib-0027] Istock, C. A. (1967). The evolution of complex life cycle phenomena: an ecological perspective. Evolution, 21(3), 592–605. 28563694 10.1111/j.1558-5646.1967.tb03414.x

[eva13477-bib-0028] Kimura, M. (1983). The neutral theory of molecular evolution. Cambridge University press.

[eva13477-bib-0029] Lande, R. (1979). Quantitative genetic analysis of multivariate evolution, applied to brain:body size allometry. Evolution, 33(1), 402–416. 28568194 10.1111/j.1558-5646.1979.tb04694.x

[eva13477-bib-0030] Lande, R. (1982). A quantitative genetic theory of life history evolution. Ecology, 63(3), 607–615.

[eva13477-bib-0031] Long, T. A. F. , Agrawal, A. F. , & Rowe, L. (2012). The effect of sexual selection on offspring fitness depends on the nature of genetic variation. Current Biology, 22(3), 204–208. 22226747 10.1016/j.cub.2011.12.020

[eva13477-bib-0032] Marshall, D. J. , Burgess, S. C. , & Connallon, T. (2016). Global change, life‐history complexity and the potential for evolutionary rescue. Evolutionary Applications, 9(9), 1189–1201. 27695526 10.1111/eva.12396 PMC5039331

[eva13477-bib-0033] Marshall, D. J. , & Keough, M. J. (2008). The evolutionary ecology of offspring size in marine invertebrates. Advances in Marine Biology, 53, 1–60. 10.1016/S0065-2881(07)53001-4 17936135

[eva13477-bib-0034] Marshall, D. J. , Krug, P. J. , Kupriyanova, E. K. , Byrne, M. , & Emlet, R. B. (2012). The biogeography of marine invertebrate life histories. Annual Review of Ecology and Systematics, 43, 97–114.

[eva13477-bib-0035] Marshall, D. J. , & Morgan, S. G. (2011). Ecological and evolutionary consequences of linked life‐history stages in the sea. Current Biology, 21(18), R718–R725. 21959162 10.1016/j.cub.2011.08.022

[eva13477-bib-0036] Martin, G. , & Lenormand, T. (2006). A multivariate extension of Fisher's geometrical model and the distribution of mutation fitness effects across species. Evolution, 60(5), 893–907. 16817531

[eva13477-bib-0037] Martin, G. , & Lenormand, T. (2008). The distribution of beneficial and fixed mutation effects close to an optimum. Genetics, 179(2), 907–916. 18505866 10.1534/genetics.108.087122 PMC2429884

[eva13477-bib-0038] Martin, G. , & Lenormand, T. (2015). The fitness effects of mutations across environments: Fisher's geometrical model with multiple optima. Evolution, 69(6), 1433–1447. 25908434 10.1111/evo.12671

[eva13477-bib-0039] McCandlish, D. M. , & Stoltzfus, A. (2014). Modeling evolution using the probability of fixation: history and implications. The Quarterly Review of Biology, 89(3), 225–252. 25195318 10.1086/677571

[eva13477-bib-0040] Metcalf, C. J. E. (2016). Invisible trade‐offs: Van Noordwijk and de Jong and life‐history evolution. The American Naturalist, 187(4), iii–v. 10.1086/685487 27028085

[eva13477-bib-0041] Moorad, J. A. , & Hall, D. W. (2009). Age‐dependent mutational effects curtail the evolution of senescence by antagonistic pleiotropy. Journal of Evolutionary Biology, 22(12), 2409–2419. 19824928 10.1111/j.1420-9101.2009.01849.x

[eva13477-bib-0042] Moorad, J. A. , & Promislow, D. E. L. (2008). A theory of age‐dependent mutation and senescence. Genetics, 179(4), 2061–2073. 18660535 10.1534/genetics.108.088526 PMC2516080

[eva13477-bib-0043] Moran, N. A. (1994). Adaptation and constraint in the complex life‐cycles of animals. Annual Review of Ecology, Evolution, and Systematics, 25(1), 573–600.

[eva13477-bib-0044] Orive, M. E. , Barfield, M. , Fernandez, C. , & Hold, R. D. (2017). Effects of clonal reproduction on evolutionary lag and evolutionary rescue. The American Naturalist, 190(4), 469–490. 10.1086/693006 28937809

[eva13477-bib-0045] Orr, H. A. (1998). The population genetics of adaptation: the distribution of factors fixed during adaptive evolution. Evolution, 52(4), 935–949. 28565213 10.1111/j.1558-5646.1998.tb01823.x

[eva13477-bib-0046] Orr, H. A. (2000). Adaptation and the cost of complexity. Evolution, 54(1), 13–20. 10937178 10.1111/j.0014-3820.2000.tb00002.x

[eva13477-bib-0047] Orr, H. A. (2005a). The genetic theory of adaptation: a brief history. Nature Reviews. Genetics, 6(2), 119–127. 10.1038/nrg1523 15716908

[eva13477-bib-0048] Orr, H. A. (2005b). Theories of adaptation: what they do and don't say. Genetica, 123(1–2), 3–13. 15881676 10.1007/s10709-004-2702-3

[eva13477-bib-0049] Orr, H. A. (2006). The distribution of beneficial fitness effects among beneficial mutations in Fisher's geometric model of adaptation. Journal of Theoretical Biology, 238(2), 279–285. 15990119 10.1016/j.jtbi.2005.05.001

[eva13477-bib-0050] Pechenik, J. A. (2006). Larval experience and latent effects – metamorphosis is not a new beginning. Integrative and Comparative Biology, 46(3), 323–333. 21672745 10.1093/icb/icj028

[eva13477-bib-0051] Pettersen, A. K. , White, C. R. , & Marshall, D. J. (2016). Metabolic rate covaries with fitness and the pace of the life history in the field. Proceedings of the Royal Society B: Biological Sciences, 283(1831), 20160323. 10.1098/rspb.2016.0323 PMC4892794 27226476

[eva13477-bib-0052] Phillips, N. E. (2002). Effects of nutrition‐mediated larval condition on juvenile performance in a marine mussel. Ecology, 83(9), 2562–2574.

[eva13477-bib-0053] Schluter, D. , Price, T. D. , & Rowe, L. (1991). Conflicting selection pressures and life‐history tradeoffs. Proceedings of the Royal Society B: Biological Sciences, 246(131), 11–17.

[eva13477-bib-0054] Sellis, D. , Callahan, B. J. , Petrov, D. A. , & Messer, P. W. (2011). Heterozygote advantage as a natural consequence of adaptation in diploids. Proceedings of the National Academy of Sciences, 108(51), 20666–20671. 10.1073/pnas.1114573108 PMC3251125 22143780

[eva13477-bib-0055] Slade, N. A. , & Wassersug, R. J. (1975). On the evolution of complex life cycles. Evolution, 29(3), 568–571. 28563189 10.1111/j.1558-5646.1975.tb00844.x

[eva13477-bib-0056] Strathmann, M. F. (1987). Reproduction and development of marine invertebrates of the northern pacific coast. The University of Washington Press.

[eva13477-bib-0057] Strathmann, R. R. (1977). Egg size, larval development, and juvenile size in benthic marine‐invertebrates. The American Naturalist, 111(978), 373–376.

[eva13477-bib-0058] Strathmann, R. R. (1993). Hypotheses on the origins of marine larvae. Annual Review of Ecology and Systematics, 24(1), 89–117.

[eva13477-bib-0059] Tenaillon, O. (2014). The utility of Fisher's geometric model in evolutionary genetics. Annual Review of Ecology, Evolution, and Systematics, 45, 179–201. 10.1146/annurev-ecolsys-120213-091846 PMC4699269 26740803

[eva13477-bib-0060] van Noordwijk, A. J. , & de Jong, G. (1986). Acquisition and allocation of resources: their influence on variation in life history tactics. The American Naturalist, 128(1), 137–142.

[eva13477-bib-0061] Wang, Z. , Liao, B. Y. , & Zhang, J. (2010). Genomic patterns of pleiotropy and the evolution of complexity. Proceedings of the National Academy of Sciences, 107(42), 18034–18039. 10.1073/pnas.1004666107 PMC2964231 20876104

[eva13477-bib-0062] Waxman, D. , & Welch, J. J. (2005). Fisher's microscope and Haldane's ellipse. The American Naturalist, 166(4), 447–457. 10.1086/444404 16224701

[eva13477-bib-0063] Werner, E. E. (1988). Size, scaling, and the evolution of complex life cycles. In B. Ebenman & L. Persson (Eds.), Size‐structured populations (pp. 27–29). Springer.

[eva13477-bib-0064] Wilbur, H. M. (1980). Complex life cycles. Annual Review of Ecology and Systematics, 11(1), 67–93.

