# Supplementary material for: Carry‐over effects and fitness trade‐offs in marine life histories: The costs of complexity for adaptation
Source: Evol Appl. 2022 Sep 20;16(2):474–85. doi: 10.1111/eva.13477 (PMC9923492; doi:10.1111/eva.13477)
Supplement: Supplementary file 1 — Figures S1‐S2 [file EVA-16-474-s001.docx]

**SUPPLEMENTARY MATERIAL**

**Distribution of mutational effects on stage-specific performance**

The effect of a mutation on performance in the *i*th stage is $\ln\left( w_{i} \right)$, where $\ln\left( w_{i} \right)>0$ for mutations that improve performance in the stage and $\ln\left( w_{i} \right)<0$ for mutations that reduce performance. Using the approximation $\gamma_{i,k}\approx{r_{i}m_{i,k}}/{\sqrt{n_{i}}}$ (see Connallon and Clark 2014b), where *m_i,k_* ~*N*(0, 1), the natural logarithm of stage-specific relative performance among mutations of magnitude *r_i_* is given by:

$$\ln\left( w_{i} \right)\approx-\omega_{i}\left( r_{i}^{2}-\frac{2r_{i}}{\sqrt{n_{i}}}\sum_{k=1}^{n_{i}} m_{i,k}\left( o_{i,k}-a_{i,k} \right) \right)$$

which is (conveniently) a linear function of standard normal random variables (the *m_i,k_* terms). Thus, mutations of size ***r*** = (*r*_1_, *r*_2_, … *r_t_*) give rise to a multivariate normal distribution of stage-specific performance effects. For the *i*th stage, the mean (averaging over the *m_i,k_* terms) is E[ln(*w_i_*) | ***r***] = –*ω_i_r_i_*^2^ and the variance is var[ln(*w_i_*) | ***r***] = (2*ω_i_z_i_r_i_*)^2^/*n_i_*, as presented in the main text.

For the set of *n* shared traits, mutational effects can be correlated between stages. For each shared trait, let $\rho_{ij}^{mut}$ represent the correlation of phenotypic effects for each shared trait expressed in stages *i* and *j* ($0<\rho_{ij}^{mut}<1$). Let $\phi_{i}={z_{i,S}}/{z_{i}}$ and $\phi_{j}={z_{j,S}}/{z_{j}}$ represent the fraction of maladaptation within each stage that is attributable to suboptimal expression of shared traits, where *z_i_* is the total displacement of the wild-type genotype from the *i*th optimum (*e.g.*, $z_{i}=\sqrt{\sum_{k=1}^{n_{i}} \left( o_{i,k}-a_{i,k} \right)^{2}}$), and *z_i,S_* is the displacement with respect to the shared traits (*e.g.*, $z_{i,S}=\sqrt{\sum_{k=1}^{n} \left( o_{i,k}-a_{i,k} \right)^{2}}$). Using the approximation $\gamma_{i,k}\approx{r_{i}m_{i,k}}/{\sqrt{n_{i}}}$, the covariance of mutational effects on performance in stages *i* and *j* is:

$$\mathrm{cov} \left[ \left. \ln\left( w_{i} \right),\ln\left( w_{j} \right) \right|\boldsymbol{r} \right]\approx\frac{4\omega_{i}\omega_{j}r_{i}r_{j}}{\sqrt{n_{i}n_{j}}}\sum_{k=1}^{n} \left( o_{i,k}-a_{i,k} \right)\left( o_{j,k}-a_{j,k} \right)\rho_{ij}=\frac{4\omega_{i}\omega_{j}z_{i}z_{j}r_{i}r_{j}}{\sqrt{n_{i}n_{j}}}\phi_{i}\phi_{j}\rho_{ij}^{mut}\cos\left( \theta_{ij} \right)$$

where $\theta_{ij}$ is the angle between vectors of directional selection to the optima of stages *i* and *j*:

$$\theta_{ij}=\cos^{-1} \left( \frac{\sum_{k=1}^{n} \left( o_{i,k}-a_{i,k} \right)\left( o_{j,k}-a_{j,k} \right)}{z_{iS}z_{jS}} \right)$$

The angle has a range of 0 < *θ_ij_* < *π*, with *θ_ij_* = 0 corresponding to a perfect alignment between the direction of selection in each stage, *θ_ij_* = *π*/2 corresponding to orthogonal orientations of selection, and *θ_ij_* = *π* corresponding to opposing directions of selection. The correlation coefficient for between-stage performance becomes:

$$\rho_{ij}^{W}=\frac{\mathrm{cov} \left[ \left. \ln\left( w_{i} \right),\ln\left( w_{j} \right) \right|\boldsymbol{r} \right]}{\sqrt{\mathrm{var} \left[ \left. \ln\left( w_{i} \right) \right|\boldsymbol{r} \right]\mathrm{var} \left[ \left. \ln\left( w_{j} \right) \right|\boldsymbol{r} \right]}}\approx\phi_{i}\phi_{j}\rho_{ij}^{mut}\cos\left( \theta_{ij} \right)$$

**Effects of isotropic residual variation on the relative fitness of each genotype**

For simplicity, and following many other applications of Fisher’s geometric model, we have assumed that an individual’s genotype specifies its phenotype in a more-or-less deterministic manner. Much of this body of theory, therefore, ignores the effects of random, residual (*e.g.*, environmental) variation on the set of phenotypes associated with a given genotype. It is worth noting that this particular assumption is not as restrictive as it first appears, provided the residual variation is isotropically distributed in each stage of the life cycle and the magnitude of the residual variation is equal among genotypes.

To see why, consider the following argument. Suppose in stage *i* that individuals with a given genotype have the following *average* phenotype:

$${\bar{\boldsymbol{A}}}_{\boldsymbol{i}}=\left( \bar{a}_{i,1},\bar{a}_{i,2},\ldots,\bar{a}_{i,n},\bar{a}_{i,n+1},\ldots,\bar{a}_{i,n_{i}} \right)$$

The optimal phenotype (as before) is:

$$\boldsymbol{O}_{\boldsymbol{i}}=\left( o_{i,1},o_{i,2},\ldots,o_{i,n},o_{i,n+1},\ldots,o_{i,n_{i}} \right)$$

Suppose that random effects of environment on the expression of each trait are drawn from a normal distribution with mean of zero and variance of *V_E_*, so that the environmental variance is isotropic in the same way that selection and mutation are isotropic in Fisher’s geometric model. The viability of an individual with the genotype in question is:

$$W_{i}\left( z_{i} \right)=\exp\left( -\sum_{k=1}^{n_{i}} \omega_{i}\left( o_{i,k}-\bar{a}_{i,k}-\varepsilon_{i,k} \right)^{2} \right)=\prod_{k=1}^{n_{i}} \exp\left( -\omega_{i}\left( o_{i,k}-\bar{a}_{i,k}-\varepsilon_{i,k} \right)^{2} \right)$$

where environmental effects per trait are IID random variables, with $\varepsilon_{i,k}\sim N\left( 0,V_{E} \right)$ representing the deviation from the genotype’s expected expression level for the *k*^th^ trait in the *i*^th^ stage.

Under these conditions, the expected viability of an individual carrying the genotype is a simple function of the mean trait values they express:

$$\bar{W}_{i}\left( \bar{z}_{i} \right)=\prod_{k=1}^{n_{i}} \int\exp\left( -\omega_{i}\left( o_{i,k}-\bar{a}_{i,k}-\varepsilon_{i,k} \right)^{2} \right)f\left( \varepsilon_{i,k} \right)d\varepsilon_{i,k}$$

where $f\left( \varepsilon_{i,k} \right)$ is the probability density function for $\varepsilon_{i,k}$, which yields:

$$\bar{W}_{i}\left( \bar{z}_{i} \right)=\prod_{k=1}^{n_{i}} \frac{1}{\sqrt{2\pi V_{E}}}\int\exp\left( -\omega_{i}\left( o_{i,k}-\bar{a}_{i,k}-\varepsilon_{i,k} \right)^{2}-\frac{\varepsilon_{i,k}^{2}}{2V_{E}} \right)d\varepsilon_{i,k}=\prod_{k=1}^{n_{i}} \frac{1}{\sqrt{2V_{E}\omega_{i}+1}}\exp\left( -\frac{\omega_{i}}{2V_{E}\omega_{i}+1}\left( o_{i,k}-\bar{a}_{i,k} \right)^{2} \right)=\left( 2V_{E}\omega_{i}+1 \right)^{{n_{i}}/2}\exp\left( -\tilde{\omega}_{i}\sum_{k=1}^{n_{i}} \left( o_{i,k}-\bar{a}_{i,k} \right)^{2} \right)$$

Fitness of a mutant individual (with mean phenotype $\bar{z}_{i}^{*}$) relative to a wild-type individual (with mean phenotype $\bar{z}_{i}$) is:

$$w_{i}=\frac{\bar{W}_{i}\left( \bar{z}_{i}^{*} \right)}{\bar{W}_{i}\left( \bar{z}_{i} \right)}=\exp\left[ -\tilde{\omega}_{i}\left( r_{i}^{2}-2\sum_{k=1}^{n_{i}} \gamma_{i,k}\left( o_{i,k}-\bar{a}_{i,k} \right) \right) \right]$$

where $\tilde{\omega}_{i}=\omega_{i}\left( 2V_{E}\omega_{i}+1 \right)^{-1}$ is an adjusted constant defining the rate at of fitness decline away from the optimum ($0<\tilde{\omega}_{i}\leq\omega_{i}$). Thus, with isotropic environmental variation in trait expression per stage, *relative* fitness per genotype takes the exact same form as it would in a model without this additional variation.”

**Effects of selection on allele frequency change across the life cycle**

At a given time, there are two alleles: a wild-type (*A*) and a mutant (*a*). We follow the frequency of the *a* allele across a series of ordered life-history stages. Let *q* represent the frequency of a before any selection has occurred. *After selection*, the frequency of *a* in stage 1 is:

$$q_{1}=\frac{qw_{1}}{1-q+qw_{1}}$$

Likewise, after selection in stages 2, and 3, the frequency of *a* will be (respectively):

$$q_{2}=\frac{q_{1}w_{2}}{1-q_{1}+q_{1}w_{2}}=\frac{qw_{1}w_{2}}{1-q+qw_{1}w_{2}}$$

$$q_{3}=\frac{q_{2}w_{3}}{1-q_{2}+q_{2}w_{3}}=\frac{qw_{1}w_{2}w_{3}}{1-q+qw_{1}w_{2}w_{3}}$$

The change in allele frequency after an arbitrary life-cycle comprised of *t* stages is:

$$\Delta q=q_{t}-q=q\left( 1-q \right)\frac{\prod_{i=1}^{t} w_{i}-1}{1-q+q\prod_{i=1}^{t} w_{i}}=\frac{sq\left( 1-q \right)}{1+qs}$$

where $s=\prod_{i=1}^{t} w_{i}-1$. Rearranging the expression for *s* gives the total fitness of the mutant (*a*) allele: $w_{a}=1+s=\prod_{i=1}^{t} w_{i}$. Invasion of a rare *a* allele is favored by natural selection when *s* > 0.

**Selection to an “effective optimum”**

With multiplicative selection across the stages, we have the total relative fitness:

$$w=\prod_{i=1}^{t} w_{i}=\prod_{i=1}^{t} \exp\left[ -\omega_{i}\left( r_{i}^{2}-2\sum_{k=1}^{n_{i}} \gamma_{i,k}\left( o_{i,k}-a_{i,k} \right) \right) \right]$$

For an individual expressing phenotype $\boldsymbol{A}_{\boldsymbol{i}}$ in the *i^th^* stage, we have:

$$W_{i}\left( \boldsymbol{A}_{\boldsymbol{i}} \right)=e^{-\omega_{i}z_{i}^{2}}=\exp\left( -\omega_{i}\sum_{k=1}^{n_{i}} \left( o_{i,k}-a_{i,k} \right)^{2} \right)$$

Total fitness for the individual becomes:

$$W\left( \boldsymbol{A} \right)=\prod_{i=1}^{t} W_{i}\left( \boldsymbol{A}_{\boldsymbol{i}} \right)=\prod_{i=1}^{t} \exp\left( -\omega_{i}\sum_{k=1}^{n_{i}} \left( o_{i,k}-a_{i,k} \right)^{2} \right)=\prod_{k=1}^{n_{i}} \exp\left( -\sum_{i=1}^{t} \omega_{i}\left( o_{i,k}-a_{i,k} \right)^{2} \right)$$

When expression of an individual’s traits is consistent across the life cycle ($a_{i,k}=a_{k}$, as expected when phenotypic effects of mutations are preserved across the life cycle, *i.e.*: *r* = *r_i_* and $\rho_{ij}^{mut}=1$), then our model can be reframed in terms of selection to an *effective* optimum. Following a similar approach to that of Cotto and Chevin (2020), we have:

$$\exp\left( -\sum_{i=1}^{t} \omega_{i}\left( o_{i,k}-a_{i,k} \right)^{2} \right)=\exp\left( -\omega_{tot}\sum_{i=1}^{t} \frac{\omega_{i}}{\omega_{tot}}\left( a_{k}-o_{i,k} \right)^{2} \right)=\exp\left( -\omega_{tot}\sum_{i=1}^{t} \left( \frac{\omega_{i}}{\omega_{tot}}a_{k}^{2}-2a_{k}\frac{\omega_{i}}{\omega_{tot}}o_{i,k}+\frac{\omega_{i}}{\omega_{tot}}o_{i,k}^{2} \right) \right)=\exp\left( -\omega_{tot}\left( a_{k}^{2}\sum_{i=1}^{t} \frac{\omega_{i}}{\omega_{tot}}-2a_{k}\sum_{i=1}^{t} \frac{\omega_{i}}{\omega_{tot}}o_{i,k}+\sum_{i=1}^{t} \frac{\omega_{i}}{\omega_{tot}}o_{i,k}^{2} \right) \right)=\exp\left( -\omega_{tot}\left( a_{k}-\tilde{o}_{k} \right)^{2}-\omega_{tot}V_{k} \right)$$

where $\omega_{tot}=\sum_{i=1}^{t} \omega_{i}$ (and, hence, $\omega_{tot}^{-1}\sum_{i=1}^{t} \omega_{i}=1$), $\tilde{o}_{k}=\sum_{i=1}^{t} \frac{\omega_{i}}{\omega_{tot}}o_{i,k}$ is the effective optimum for the *k^th^* trait, and $V_{k}=\sum_{i=1}^{t} \frac{\omega_{i}}{\omega_{tot}}o_{i,k}^{2}-\left( \sum_{i=1}^{t} \frac{\omega_{i}}{\omega_{tot}}o_{i,k} \right)^{2}$ is the effective variance among stages for the *k^th^* trait optimum.

Substituting these expressions into the general equation for $W\left( \boldsymbol{A} \right)$, we obtain:

$$W\left( \boldsymbol{A} \right)=\prod_{k=1}^{n_{i}} \exp\left( -\omega_{tot}\left( a_{k}-\tilde{o}_{k} \right)^{2}-\omega_{tot}V_{k} \right)=\exp\left( -\omega_{tot}\sum_{k=1}^{n_{i}} V_{k} \right)\exp\left( -\omega_{tot}\sum_{k=1}^{n_{i}} \left( a_{k}-\tilde{o}_{k} \right)^{2} \right)$$

The fitness of mutant relative to wild-type individuals becomes:

$$w=\frac{W\left( \boldsymbol{M} \right)}{W\left( \boldsymbol{A} \right)}=\exp\left( -\omega_{tot}r^{2}+\omega_{tot}2r\sum_{k=1}^{n_{i}} \frac{m_{k}}{\sqrt{\sum_{k=1}^{n_{i}} m_{k}^{2}}}\left( \tilde{o}_{k}-a_{k} \right) \right)$$

With high dimensionality and small fitness effects of mutations on relative fitness, we have:

$$\log\left( 1+s \right)\approx s\approx-\omega_{tot}r^{2}+\frac{2\omega_{tot}r}{\sqrt{n}}\sum_{k=1}^{n_{i}} m_{k}\left( \tilde{o}_{k}-a_{k} \right)$$

$$\bar{s}\approx-\omega_{tot}r^{2}$$

$$\sigma^{2}\approx\frac{\left( 2\omega_{tot}\tilde{z}r \right)^{2}}{n}$$

where $\tilde{z}=\sum_{k=1}^{n_{i}} \left( \tilde{o}_{k}-a_{k} \right)^{2}$ is the population’s distance from the effective optimum within the multivariate phenotypic space.

**Trade-offs in performance across life-history stages**

In a life cycle comprised of *t* stages, the probability that a mutation is unequivocally deleterious is given by:

Pr(deleterious) = Pr(ln(*w*_1_), ln(*w*_2_), …, ln(*w_t_*) > 0)

The probability that a mutation is beneficial in at least one life-history stage is:

1 – Pr(ln(*w*_1_), ln(*w*_2_), …, ln(*w_t_*) > 0)

Among mutations that are beneficial in at least one stage, the fraction that exhibit a trade-off with any other stage is:

$$f_{A}=\frac{1-\Pr\left( \ln\left( w_{1} \right),\ln\left( w_{2} \right), \ldots,\ln\left( w_{t} \right)<0 \right)-\Pr\left( \ln\left( w_{1} \right),\ln\left( w_{2} \right), \ldots,\ln\left( w_{t} \right)>0 \right)}{1-\Pr\left( \ln\left( w_{1} \right),\ln\left( w_{1} \right), \ldots,\ln\left( w_{t} \right)<0 \right)}$$

While there is no general solution to this equation, we can define a lower bound for *f_A_* for special cases of our model. Assuming that mutational effects in each stage are weak (*r_i_* 🡪 0), dimensionality is modest-to-high (*n* > ~10), and the variance for mutational effects on performance, var(ln(*w_i_*)|***r***), is equal among stages, then the distribution of mutational effects on performance across the set of stages is approximately multivariate normal with a mean near the origin: E(ln(*w_i_*)|***r***) ~ (0, 0, … , 0). In this weak mutation-limit (and similar to the limit in the original version of Fisher’s geometric model; Fisher 1930), the relative fractions of mutations that are unequivocally beneficial and unequivocally deleterious will be approximately equal (*i.e.*: Pr(ln(*w*_1_), ln(*w*_2_), …, ln(*w_t_*) < 0) ≈ Pr(ln(*w*_1_), ln(*w*_2_), …, ln(*w_t_*) > 0), and the remaining fraction of mutations exhibits performance trad-offs between stages.

Positive and negative quadrants for the bivariate standard normal distribution are given by:

$$\Pr\left( y_{1},y_{2}<0 \right)=\Pr\left( y_{1},y_{2}>0 \right)=\frac{1}{4}+\frac{1}{2\pi}\arcsin\left( \rho_{12} \right)$$

where *y*_1_ and *y*_2_ are standard normal random variables with correlation of *ρ*_12_ (Rose and Smith 2002, pp. 231). By extension with two stages in our model, and under the weak-mutation assumptions outlined above, we have:

$$\Pr\left( \left. \ln\left( w_{1} \right),\ln\left( w_{2} \right)>0 \right|\boldsymbol{r} \right)\approx\Pr\left( \left. \ln\left( w_{1} \right),\ln\left( w_{2} \right)<0 \right|\boldsymbol{r} \right)\approx\frac{1}{4}+\frac{1}{2\pi}\arcsin\left( \rho_{12}^{w} \right)$$

and (therefore):

$$\Pr\left( \left. \ln\left( w_{1} \right)>0>\ln\left( w_{2} \right) \right|\boldsymbol{r} \right)\approx\Pr\left( \left. \ln\left( w_{2} \right)>0>\ln\left( w_{1} \right) \right|\boldsymbol{r} \right)\approx\frac{1}{4}-\frac{1}{2\pi}\arcsin\left( \rho_{12}^{w} \right)$$

The fraction of conditionally beneficial mutations that trade-off with the other stage will be:

$$f_{A}\approx\frac{2\pi-4\arcsin\left( \rho_{12}^{w} \right)}{3\pi-2\arcsin\left( \rho_{12}^{w} \right)}$$

which simulations confirm represents a lower bound (see Fig. 2 in the main text).

We can obtain analogous results for the 3-stage case by first noting that the positive and negative octants for the tri-variate standard normal distribution are:

$$\Pr\left( y_{1},y_{2},y_{3}<0 \right)=\Pr\left( y_{1},y_{2},y_{3}>0 \right)=\frac{1}{8}+\frac{\arcsin\left( \rho_{12} \right)+\arcsin\left( \rho_{13} \right)+\arcsin\left( \rho_{23} \right)}{4\pi}$$

where *y*_1_, *y*_2_, and *y*_3_ are tri-variate standard normal random variables with pairwise correlations of *ρ*_12_, *ρ*_13_, and *ρ*_23_ (Rose and Smith 2002, pp. 231). Applied to the case of three stages in our model, we have:

$$\Pr\left( \left. \ln\left( w_{1} \right),\ln\left( w_{2} \right),\ln\left( w_{3} \right)>0 \right|\boldsymbol{r} \right)\approx\Pr\left( \left. \ln\left( w_{1} \right),\ln\left( w_{2} \right),\ln\left( w_{3} \right)<0 \right|\boldsymbol{r} \right)\approx\frac{1}{8}+\frac{\arcsin\left( \rho_{12}^{w} \right)+\arcsin\left( \rho_{13}^{w} \right)+\arcsin\left( \rho_{23}^{w} \right)}{4\pi}$$

and (therefore):

$$f_{A}\approx\frac{6\pi-4\left( \arcsin\left( \rho_{12}^{w} \right)+\arcsin\left( \rho_{13}^{w} \right)+\arcsin\left( \rho_{23}^{w} \right) \right)}{7\pi-2\left( \arcsin\left( \rho_{12}^{w} \right)+\arcsin\left( \rho_{13}^{w} \right)+\arcsin\left( \rho_{23}^{w} \right) \right)}$$

As with the bivariate case, this approximation gives a lower bound (see Fig. 2, main text).

**Rates of adaptive evolution**

Following the general approach of Orr (2000; also see Tenaillon et al. 2014; Connallon and Hall 2018), the rate of adaptation in the haploid version of Fisher’s geometric model is:

$$R=2Nu\int_{0}^{\infty} s^{2}f\left( s \right)ds$$

where *f*(*s*) is the probability density function for the fitness effects of mutations. At sufficiently high dimensionality (*e.g.*, *n* > 10), the selection coefficients (*s*) among mutations with magnitude ***r*** = (*r*_1_, *r*_2_, … *r_t_*) are approximately normally distributed with a mean and variance given by eqs (9) and (10) in the main text. Substituting $f\left( s \right)=\frac{1}{\sqrt{2\pi\sigma^{2}}}\exp\left( -\frac{\left( s-\bar{s} \right)^{2}}{2\sigma^{2}} \right)$, the rate of adaptation via mutations of magnitude ***r*** becomes:

$$R=2Nu\int_{0}^{\infty} s^{2}\frac{1}{\sigma\sqrt{2\pi}}\exp\left( -\frac{\left( s-\bar{s} \right)^{2}}{2\sigma^{2}} \right)ds=Nu\sigma^{2}\left[ \left( 1+\frac{\bar{s}^{2}}{\sigma^{2}} \right)\left( 1+\mathrm{erf} \left( \frac{\bar{s}}{\sigma\sqrt{2}} \right) \right)+\frac{\bar{s}}{\sigma}\frac{\sqrt{2}}{\sqrt{\pi}}e^{-{\bar{s}^{2}}/{2\sigma^{2}}} \right]$$

as presented in the main text.

**Supplementary References**

Rose C, Smith MD. 2002. *Mathematical Statistics with Mathematica*. Springer-Verlag, New York.

**Figure S1.** Stage-specific adaptation and orientations of directional selection during adaptive walks toward stage-specific optima: further results for the case where the phenotypic effects of mutations are strongly correlated among stages (*r*_1_ = *r*_2_ = *r*_3_; $\rho_{ij}^{mut}=1$). The left-hand panel shows the scenario where optima of the three stages are equally distant from one another (initial conditions: *z*_1_ = *z*_2_ = *z*_3_ = 1; cos(*θ*_12_) = cos(*θ*_13_) = cos(*θ*_23_) = 0.95; this scenario is analogous to the top panel Fig. 3 in the main text). The central and right-hand panels show scenarios where the optima for stages 2 and 3 are identical, and the optimum for stage 1 is divergent from the others (initial conditions: *z*_1_ = *z*_2_ = *z*_3_ = 1; cos(*θ*_12_) = cos(*θ*_13_) = 0.95; cos(*θ*_23_) = 1). Carry-over effects are present in the right-hand panel (where *c_ij_* > 0), and absent in the other two (where *c_ij_* = 0). The averages (the black and red curves) are based on 500 simulated adaptive walks. Correlations between the orientations of directional selection in stages 1 and 2—which are captured by $\cos\left( \theta_{12} \right)$, where $\theta_{12}$ is the angle between vectors of multivariate directional selection in the two stages—are shown for the first 50 adaptive walks (each in gray). Results show cases where *ω*_1_ = *ω*_2_ = *ω*_3_ = ½, with mutational magnitudes (*r*_1_, *r*_2_, *r*_3_) following an exponential distribution (gamma with parameters *α* = 1 and *λ* = 0.1).

**
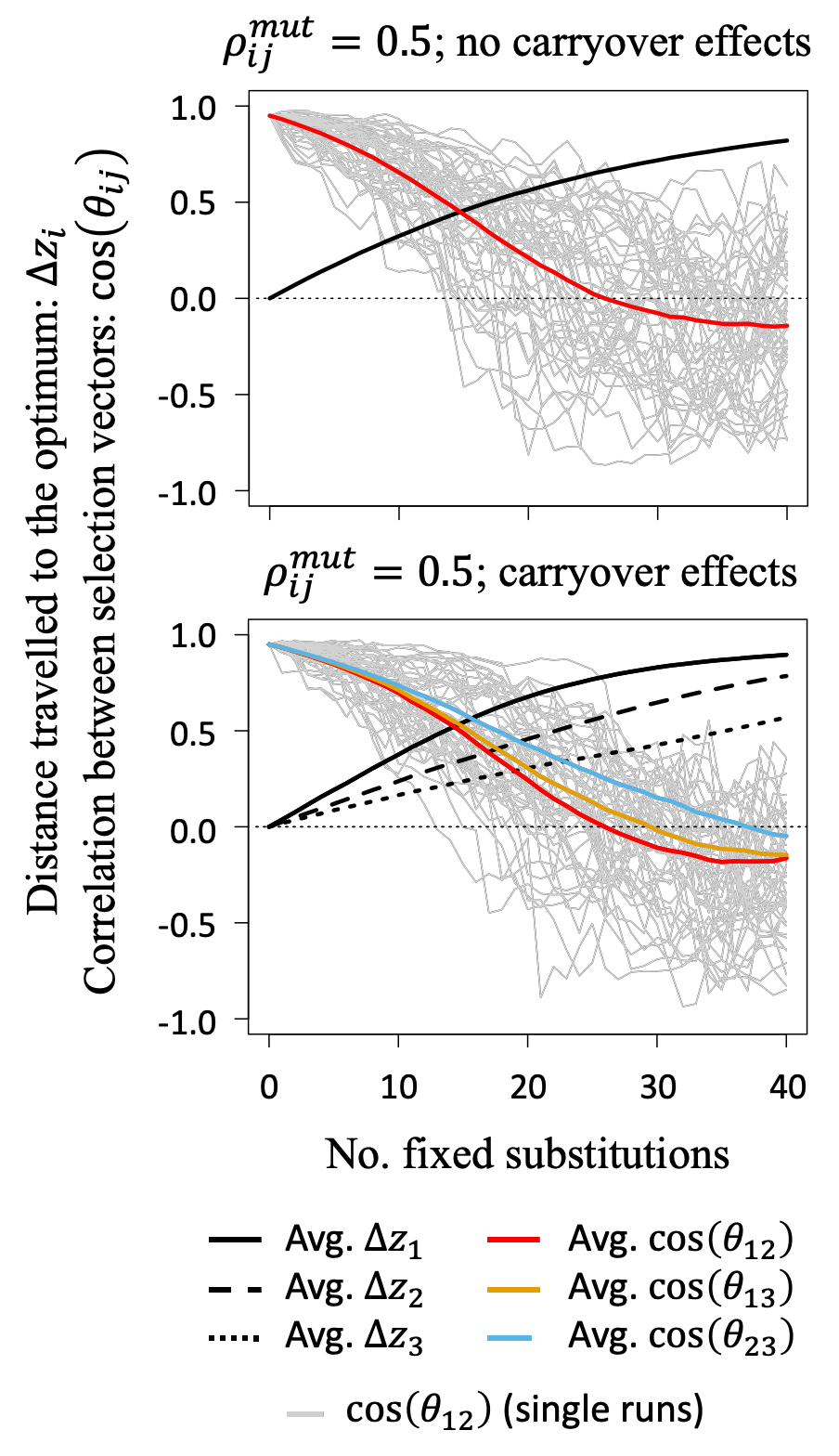
**

**Figure S2.** Stage-specific adaptation when mutant phenotypic effects exhibit intermediate correlations among stages. Results apply to the same scenarios as presented in Figs. 3 and 4 of the main text, but with intermediate mutant phenotypic effect correlations between an arbitrary pair of stages *i* and *j*. Results represent the case where of a correlation coefficient of 0.5 for the overall phenotypic effect magnitude in each stage (corr(*r_i_*, *r_j_*) = 0.5) and mutational effects on individual shared traits ($\rho_{ij}^{mut}=0.5$).
